# Expressive language sampling as a source of outcome measures for treatment studies in fragile X syndrome: feasibility, practice effects, test-retest reliability, and construct validity

**DOI:** 10.1186/s11689-020-09313-6

**Published:** 2020-03-24

**Authors:** Leonard Abbeduto, Elizabeth Berry-Kravis, Audra Sterling, Stephanie Sherman, Jamie O. Edgin, Andrea McDuffie, Anne Hoffmann, Debra Hamilton, Michael Nelson, Jeannie Aschkenasy, Angela John Thurman

**Affiliations:** 1 UC Davis MIND Institute and Department of Psychiatry and Behavioral Sciences, University of California, 2825 50th St. Davis, Sacramento, CA 95817 USA; 2 Departments of Pediatrics, Neurological Sciences and Biochemistry, Rush University Medical Center, Chicago, USA; 3 Waisman Center and Department of Communication Sciences and Disorders, University of Wisconsin-Madison, Madison, USA; 4 Department of Human Genetics, Emory University, Atlanta, USA; 5 Department of Psychology, University of Arizona, Tucson, USA

**Keywords:** Outcome measures, Clinical trials, treatment, Expressive language, Fragile X syndrome, Psychometrics

## Abstract

**Background:**

The evaluation of treatment efficacy for individuals with fragile X syndrome (FXS) or intellectual disability (ID) more generally has been hampered by the lack of adequate outcome measures. We evaluated expressive language sampling (ELS) as a procedure for generating outcome measures for treatment research in FXS. We addressed: (a) feasibility, (b) practice effects over two administrations, (c) test-retest reliability over the repeated administrations, and (d) construct validity. We addressed these issues for the full sample as well as for subgroups defined by age, IQ, and ASD status.

**Methods:**

Participants were 106 individuals with FXS between ages 6 and 23 years who had IQs within the range of intellectual disability (IQ < 70). ELS procedures for collecting samples in conversation and narration were followed and analyzed separately. Five measures were derived from transcripts segmented into C-units (i.e., an independent clause and its modifiers): number of C-units per minute (talkativeness), number of different word roots (vocabulary), C-unit length in morphemes (syntax), percentage of C-units containing dysfluency (utterance planning), and percentage of C-units that were fully or partly unintelligible (articulatory quality). ELS procedures were administered twice at 4-week intervals for each participant. Standardized tests and informant reports were administered and provided measures for evaluating construct validity of ELS measures.

**Results:**

We found low rates of noncompliance, suggesting the task can be completed meaningfully by most individuals with FXS, although noncompliance was higher for younger, lower IQ, and more autistic participants. Minimal practice effects and strong test-retest reliability over the 4-week interval were observed for the full sample and across the range of ages, IQs, and autism symptom severity. Evidence of convergent construct validity was observed for the measures of vocabulary, syntax, and unintelligibility for the full sample and across the range of IQ and autism symptom severity, but not for participants under age 12. Conversation and narration yielded largely similar results in all analyses.

**Conclusions:**

The findings suggest that the ELS procedures are feasible and yield measures with adequate psychometric properties for a majority of 6 to 23 years with FXS who have ID. The procedures work equally well regardless of level of ID or degree of ASD severity. The procedures, however, are more challenging and have somewhat less adequate psychometric properties for individuals with FXS under age 12.

## Background

Approximately 1 in every 77 children in the USA has an intellectual disability (ID) [1]. Until recently, the etiology was unknown for most cases of ID [2]. Advances in genomics, however, have now identified more than 100 genes that have a causal role [3] and that collectively account for half of all cases of ID [4]. Moreover, research spanning multiple levels of analysis, from the cellular to the behavioral, has uncovered both commonalities and differences in the phenotypes of conditions resulting from disparate etiologies, such as fragile X syndrome (FXS) and Down syndrome [5]. This research also has led to etiology- or disorder-specific treatments, with dozens of pharmaceuticals already tested in clinical trials [6–8] and more trials planned. Etiology-specific behavioral, educational, and psychosocial treatments are also emerging [4, 9, 10]. Although treatments are being developed for a range of disorders associated with ID, the testing of pharmacological treatments for FXS has arguably been the most active [11]. FXS is an X-linked single-gene disorder [12] and is the leading inherited cause of ID with a prevalence of 1 in 3600 males and 1 in 4000 to 6000 females [13–15].

Despite the growth of clinical trials in ID research, evaluation of treatment efficacy in individuals with FXS [16] and other ID conditions [7, 17], however, has been hampered by the lack of adequate outcome measures. In the case of FXS, more than two dozen clinical trials testing a range of promising drugs have largely failed to show efficacy in humans [11]. Failed trials have been the norm for other ID conditions as well [18]. The lack of appropriate outcome measures for individuals with FXS and other ID conditions has been viewed as one of the most important contributors to the failure of these trials [7, 16, 19]. Many of these trials have relied on parent report measures, which were then found to be subject to large placebo effects that may have obscured benefits of the drugs being studied [20, 21]. Although standardized tests would appear to offer a more direct and objective assessment of change, these tests have not been validated for FXS, and thus, their utility in clinical trials is unclear [22]. In fact, closely spaced, repeated administrations of most standardized tests, as required in a clinical trial, are discouraged because of concerns about practice effects and the short-term instability of scores [22]. In addition, individuals with ID often score at the floor of standardized tests, making it difficult to assess differences at baseline or the magnitude of change among individuals [23, 24]. Finally, there are concerns about the extent to which standardized tests predict an individual’s functioning in real-world contexts that are meaningful to the individual as required by the Food and Drug Administration [19, 22].

The development of outcome measures is complicated by the heterogeneity observed across individuals with ID. Even within a single etiological condition there is considerable heterogeneity [4, 20–23]. In FXS, for example, IQ can range from the typical range to the more severe end of the ID range [24–26]; about half of affected individuals meet diagnostic criteria for autism spectrum disorder (ASD [27];); and indicators of affectedness vary with age, such as seen in the decline in IQ during adolescence [28]. This heterogeneity makes it difficult to find a single measure that can be appropriate for characterizing all, or even most, individuals with FXS. Unfortunately, the designation of a single outcome measure as primary is required for deciding on the efficacy of the drug in these trials [29]. Thus, there is a pressing need for psychometrically adequate outcome measures that have a wide range of applicability in terms of participant characteristics.

### Requirements for outcome measures

There have been several publications, some emerging from recent NIH-convened working groups, that have outlined the requirements for adequate outcome measures for treatment studies in the ID field [7, 16, 17, 19, 25]. In general, there has been a push for outcome measures that are directly administered to the participant rather than relying on informant report, especially when the informant is a parent or care provider, because of the susceptibility of such measures to placebo effects [29], although there are ongoing attempts to create informant-report measures that are resistant to such effects.

In terms of directly administered measures, several requirements have been cited [22]. First, an outcome measure must be feasible for the target population. This requirement implies (a) a range of difficulty that maps onto the ability levels characterizing the disorder, thereby ensuring the measure is not subject to floor or ceiling effects and (b) that the measure is sufficiently motivating for the target populations so that noncompliance rates are low. Second, the measure must be subject to limited practice effects (i.e., significant improvements or decrements) over repeated administration. Practice effects may limit the potential for additional treatment-induced change or may, in some research designs, make it difficult to apportion change to practice versus treatment. Third, the measure should be reliable over repeated administrations, which is typically evidenced by high correlations between repeated administrations on the same sample of participants. Put differently, the participants should maintain their rank order of scores and with the same absolute magnitude of differences among them on each administration of the measure. Fourth, there should be evidence of the construct validity of the measure (e.g., the measure should correlate with other measures of the same ability or attribute) in the target population. In other words, it must be clear that the measure reflects the skill, ability, or attribute that is intended for the target population. Fifth, the measure should be linked to real-world functioning for the target population, so that any change on the measure within the context of a treatment study is meaningful for the individual. Finally, the measure must be sensitive to any change in the intended construct that is produced by the treatment in question. Of course, analyzing the sensitivity to change in a measure makes little sense in advance of demonstrating that the measure meets the other criteria listed above.

### Expressive language sampling

In the present study, we evaluated expressive language sampling (ELS) for its adequacy in generating outcome measures for treatment research in FXS. In ELS, samples of the participant’s talk are collected in naturalistic interactions with another person [30]. In the specific ELS procedures we have developed, the partner is an examiner and the format, content, and behavior of the examiner are scripted to ensure reasonable consistency of sampling context across participants, occasions of measurement, and examiners [31, 32]. At the same time, however, the interactions remain naturalistic and the examiner is able to adapt to the interests and behavior of the participant (within constraints) so as to enable generalizability to real-world social interaction [33]. Audio-recordings of these samples are transcribed and then analyzed using computer-based algorithms to derive clinical endpoints reflecting several dimensions of language ability [32, 34].

ELS procedures of various sorts have been used for decades in research and clinical practice to diagnose language impairments and track developmental changes in language skills; however, none of these procedures has been fully validated for studies of treatment efficacy in FXS or other ID conditions [7, 16]. A focus on language as an endpoint for treatment studies, however, is particularly attractive given the critical role of language for full participation in the community and for acquiring other adaptive skills, and thus, treatments that improve language are likely to be meaningful for the individual [35]. In addition, expressive language impairments are ubiquitous among individuals with ID [36], and the profile of relative strengths and weaknesses across different dimensions of expressive language distinguishes among many of the syndromes associated with ID [9].

ELS has several potential advantages compared to typical norm-referenced standardized tests [30], such as the Receptive and Expressive subscales of the Mullen Early Scales [37], the Peabody Picture Vocabulary Test-4th edition [38], the Preschool Language Scale-5 [39], the Clinical Evaluation of Language Fundamentals [40], and the Comprehensive Assessment of Spoken Language [41], all of which have been used in clinical trials for FXS [42–45]. First, ELS interactive contexts are more closely aligned with performance in real-world contexts, and thus, performance is more likely to be generalizable to activities that are functional and meaningful for the individual [46]. Second, numerous dependent measures, each reflective of a different dimension of language, can be computed from a single expressive language sample [30, 31], which allows the selection of a single focused clinical endpoint rather than simply the omnibus language score(s) on most standardized tests [32]. Third, many ELS interactive contexts can be used with both children and adults and have low rates of noncompliance and limited floor effects for individuals producing at least some multiword utterances [30, 33]. Fourth, ELS measures are better than standardized tests in discriminating typically developing (TD) children from clinically identified children with specific language impairment (SLI [47–50], especially for children from ethnic and racial minority groups [51]. Finally, intervention studies targeting children with ASD [52, 53] and children and adolescents with FXS have shown change in various ELS-type procedures in the face of a lack of change in standardized tests.

Measures derived from various ELS procedures have strong psychometric properties for a number of populations. For example, Heilmann et al. [54] found moderate, but statistically significant, test-retest reliabilities for multiple measures of vocabulary computed from 4-min long samples collected from kindergarten through second grade-aged children learning English as a second language. In terms of construct validity, measures derived from ELS reflecting syntactic complexity, vocabulary, and talkativeness correlate with age, nonverbal cognitive level, and other language measures for TD children [49, 55–58]. Comparable data on ID populations, such as FXS, however, are very limited. In the proposed project, therefore, we will evaluate the validity of these dependent measures computed from expressive language samples collected from individuals with FXS.

The psychometric properties of ELS as a source of outcome measures for FXS have been examined in only a single small-scale pilot study, albeit with promising results. Berry-Kravis, Doll et al. [32] administered ELS procedures to 36 participants with FXS (25 males), ages 5 to 36 years, all of whom produced at least some multiword combinations according to parent report. The investigators used the ELS procedures for collecting samples in two contexts: narration of a wordless picture book and conversation on a predetermined set of topics. They used two contexts because there is considerable evidence that some aspects of language vary consistently with contextual factors. For example, conversation tends to elicit a greater breadth of vocabulary, whereas narration tends to elicit more syntactically complex language [31]. ELS procedures were administered twice to each participant (2 to 3 weeks apart) to assess test-retest reliability, with a different version of the materials used for test and retest per participant. Construct validity was evaluated by correlating the dependent measures from the ELS narration and conversation samples with the Expressive Communication domain raw scores from the Vineland Adaptive Behavior Scales—Second Edition (VABS-II) [59].

Berry-Kravis, Doll et al. [32] found that all the participants were able to meaningfully complete the narration and conversation tasks. They examined test-retest reliability and construct validity for five dependent measures for each task, with the measures designed to capture a broad array of expressive language skills: talkativeness, utterance planning, articulation accuracy, vocabulary, and syntax. Practice effects were minimal, with only the utterance planning measure in conversation showing statistically significant, but small, improvement from test to retest administrations. Intraclass correlation coefficients between the test and retest administrations were all significant and ranged from .91 to .97 across measures for conversation and from .73 to .94 across measures for narration. Bivariate correlations between the ELS dependent measures (except for talkativeness) were significantly correlated with the Expressive Communication domain raw scores from the VABS-II, although only syntax was correlated for both conversation and narration. Thus, these pilot data provide preliminary support for the promise of the ELS-derived measures.

The Berry-Kravis, Doll et al. study [32], although promising in its findings, has a number of limitations that the present study was designed to overcome. First, the sample size was relatively small, which raises concerns about generalizability of findings as well as the possibility that the study was statistically under-powered to detect practice effects. Second, the sample for the study was recruited from a single clinical site, and the ELS procedures were administered by a single examiner, which raises concerns about the generalizability of the findings to the multi-site, multi-examiner format that is more typical of clinical trials in the ID field. Third, the measure used for construct validity (i.e., the Expressive Communication domain score from the VABS-II) is rather broad, tapping more than language (e.g., it includes questions about writing) and does not provide separate subscale scores that could be expected to map differentially onto the different dependent measures derived from ELS. Thus, the VABS-II and ELS constructs are only partly overlapping and provide no basis for exploring both convergent and discriminant validity. Fourth, the sample size precluded any meaningful investigation of variation in the psychometric properties of the ELS measures as a function of participant characteristics, which would be critical information for the determination of eligibility criteria for any clinical trials using ELS-derived outcome measures.

In this article, we report on the initial results of an ongoing multi-site study of the psychometric properties of ELS-derived measures for a large sample of US, English-speaking individuals with FXS. Participants varied in age from young school-age children to young adults. All participants had IQs in the range of ID, and all were verbal and capable of producing some multiword combinations. In the analyses reported here, we addressed the following: (1) feasibility, (2) practice effects over two administrations, (3) test-retest reliability over the repeated administrations, and (4) construct validity via correlations with other non-ELS language measures. We addressed these issues for the sample as a whole, as well as, in exploratory analyses, subgroups defined by age, IQ, and ASD status to understand the relationships among these individual difference dimensions and the measure properties.

## Method

### Participants

Participants were recruited between a chronological age (CA) of 6 and 23 years at first testing. We adopted age 6 years as a minimum based on expectations concerning the limited capacity of children with ID under 6 to complete the ELS procedures (Abbeduto et al. [37]), their frequent lack of sufficient phrase speech, and their relatively infrequent inclusion in clinical trials to date. We adopted 23 years as the upper age to increase the likelihood of including individuals who lived at home with a parent to facilitate recruitment of adults. In addition, a parallel arm of the project focused on Down syndrome and limiting the sample to young adults decreased the likelihood of dementia in our Down syndrome group. In addition to Down syndrome, the larger project from which the present data are drawn, also included individuals with ASD. Data from these other groups will be reported in subsequent papers.

Participants met the following criteria (according to parent/guardian report): (1) speech is the primary mode of communication, (2) produces at least occasional three-word or longer utterances, (3) English is the primary language spoken in the home, (4) no more than a mild hearing loss, and (5) no serious (uncorrected) visual impairments that would preclude successful performance on the testing battery. Participants also had IQs within the range of intellectual disability (IQ < 70), first determined through parent report and record review, and subsequently confirmed via direct testing in the study (described below). We also required confirmation of the *FMR1* full mutation (i.e., CGG repeats > 200) through previous molecular DNA testing (PCR and Southern blot) determined by a medical report provided by parents/guardians or (with permission) from medical records. The Institutional Review Boards of all the universities participating in the project provided approval, with parent/guardian written informed consent obtained for all participants prior to testing.

We tested 106 individuals with FXS with ID across four of the participating university sites, which included 8 pairs of siblings. The sample was, as expected, predominantly male (*n* = 85). The sample was predominantly white, but 25% of the sample identified as non-white, Hispanic, or multi-racial. We had hoped to recruit equal numbers of participants in the age groups of 6 to 11 years, 12 to 17 years, and 18 to 23 years; however, we were more successful recruiting for the middle group (*n* = 55), achieving samples of 28 and 23 in the youngest and oldest age groups, respectively. Additional participant characteristics are provided in Table 1.

**Table 1:** Participant characteristics

Measure	***M***	SD	Range
Chronological age	14.8	4.6	6.5–23.8
NVIQ^a,c^	46.4	6.2	42–68
VIQ^a,d^	48.1	7.2	42–78
Full scale IQ^a,c^	44.8	6.7	40–66
ADOS severity^b,e^	5.7	2.4	1–10

*n* = 106 unless indicated otherwise

^a^Stanford-Binet, 5th edition standard scores

^b^Severity score from Autism Diagnostic Observation Schedule, 2nd edition

^c^*n* = 103

^d^*n* = 105

^e^*n* = 100

Once enrolled, we required that participants could not also be enrolled in a randomized clinical trial during, or for the 8 weeks prior to, the initial testing visit or during the period between the initial testing and the retesting visit, which was 4 weeks later on average. Although medications to manage behavior prescribed by a physician (e.g., SSRIs), or participation in an Open Label clinical trial, were allowed, we excluded or tried to reschedule any participant experiencing a change in these medications between the first and retest visits or in the 8 weeks before the initial testing visit. Medication use was determined by parent/guardian report. We also excluded or tried to reschedule participants who (according to parent/guardian report) had a major change in behavioral therapy or educational programming between the first and retest visits or in the 8 weeks preceding the initial testing visit, with regular school vacations not considered to be a programming change.

### Measures

The following measures were administered on an individual basis to participants with the aims being (a) to characterize the degree of impairment of participants and (b) to create variables for examining the psychometric properties of ELS in relation to important individual difference variables or to establish the construct validity of the ELS measures. These measures were administered on the same day as the first administration of the ELS procedures for 86% of our sample; the remaining participants completed the two halves of the study visit on different days, with all but 1 completing the visits within 8 days or less.

### Intellectual functioning

The *Stanford-Binet Intelligence Scales, Fifth Edition* ((SB5) Roid, 2003) [60] were administered. The SB5 is comprised of 10 subtests that cover the age range of 2 to 89+ years. The SB5 yields a nonverbal, verbal, and full-scale IQ. Mean IQ = 100, SD = 15. These scores were used to determine eligibility for the study and provide a description of the participants (see Table 1). Given the significant floor effects observed on the SB5 in individuals with FXS, we also utilized the Sansone et al. [23] method of raw *z*-score transformation (based on the general population norms). This method has been shown to ameliorate floor effects and improve the precision of IQ scores for individuals with FXS. The transformed nonverbal, verbal, and full-scale mean scores (and ranges) for the sample were 46.6 (1.4, *n* = 96), 46.0 (1.5, *n* = 101), and 46.9 (1.4, *n* = 93), respectively. The lack of participant compliance or examiner errors in administration (e.g., failure to establish a ceiling) led to missing values for a few participants.

### ASD symptom severity

We administered the Autism Diagnostic Observation Schedule, 2nd edition (ADOS-2) according to the standard procedures [61]. The ADOS-2 is comprised of a series of activities that provide the opportunity to observe behaviors reflecting the core impairments of ASD. The ADOS-2 has four modules, each designed for individuals with different degrees of impairment and verbal skills. The module administered to any given participant was selected according to the ADOS-2 manual guidelines. The number of participants receiving each module was 11, 40, and 49 for modules 1, 2, and 3, respectively. Because of the level of ID and relative lack of independence demonstrated by the participants in the present study, none met ADOS-2 manual guidelines for administration of the module 4. The ADOS-2 was administered by a research-reliable examiner, who scored the participant’s behavior in real time. Errors in examiner administration, participant noncompliance, and scheduling difficulties resulted in missing values on this measure for six of the 106 participants.

### Expressive language sampling

Expressive language samples for the present study were collected twice from each participant (test and retest), with a target interval of 4 weeks between the two administrations (*M* = 29.7 days, SD = 6.5, range = 19–55 days). Deviations from the 4-week window reflected scheduling difficulties and accommodation to treatment changes. At each time point, samples were taken in two contexts—conversation and narration—with the order of administration randomized across participants. The particular conversation and narration procedures used have been used in several other studies [31, 32, 34, 62–65]. In general, the procedures were designed to be naturalistic while ensuring reasonable standardization of materials, content of the talk, and examiner behavior. Such standardization is critical because of the considerable research demonstrating that variation in such domains can have dramatic influences on children’s language output [30]. In the final sample of participants, 53 received conversation before narration at test and 53 received conversation before narration at retest. In the larger project, we also are analyzing the ADOS interaction to determine the psychometrics of that measure from the perspective of a language sampling tool, but those data will be presented in subsequent papers.

In conversation, the examiner engages the participant in talk on a variety of topics (e.g., school, family, hobbies) according to guidelines that specify the order of topics and the ways in which topics are introduced and maintained. The conversation begins with a topic that the parent/guardian has previously indicated is one of several that the participant would enjoy sharing, thereby ensuring maximum comfort with the interaction and avoiding any topics that could lead to frustration. Idiosyncratic topics for the participants in the present sample included preferred activities (e.g., eating at a restaurant, playing video games, and sports), fictional characters and famous people (e.g., Iron Man, Elmo, and Tim McGaw), familiar people (e.g., family members, classmates, friends), specific events and activities (e.g., a particular flight, a specific visit to the beach), and specific topics (e.g., trains, weather, country music). If a participant perseverates on an idiosyncratic topic, the examiner waits for a natural stopping point to introduce the next topic, but with no more than about 3 min allowed on the idiosyncratic topic. The remaining “standard” topics are selected in order from a predetermined list. In general, the script that the examiner follows minimizes his/her participation, maximizes the participant’s contribution, and avoids frequent use of examiner language that would constrain the amount or complexity of participant talk (e.g., yes-no questions). In preliminary work, we found it necessary to use slightly different sets of topics for children/adolescents relative to adults (e.g., school is a useful topic for the former, but not the latter). The procedures are otherwise identical for participants of different ages. The conversation is ideally brought to a close by the examiner after 12 min.

We also created two sets of topics (versions A and B) for children/adolescents and two for adults, which made it possible to present alternate versions in test and retest administrations for any given participant. The scripts for the two versions are otherwise identical. Assignment of version to test and retest was randomly determined across participants, with 50 of the 106 participants receiving version A at the first visit. A participant who received version A at test received version B at retest and vice versa.

In narration, the participant tells the story in a wordless picture book. Examiner prompts and responses are scripted. The procedure begins with the examiner asking the participant to look at the book to get a sense of the story, but without talking about it. The examiner controls the turning of the pages so that the participant reviews each pair of pages for 8 to 10 s. The participant then tells the story page by page, with page turning controlled by the examiner. As in conversation, the examiner follows a script that minimizes his/her participation, maximizes the participant’s contribution, and avoids the use of examiner language that would constrain the participant’s talk. The administration is untimed but typically takes 10 to 15 min to administer and yields narratives of 3 to 8 min in length for TD children (Kover et al. [40]).

We used two books from Mercer Mayer’s “frog” series (*Frog goes to Dinner* and *Frog, Where Are You?*). We found previously that these two books yield expressive language samples that do not differ on the dependent variables of interest for individuals with FXS [34], making it possible to present alternate versions in test and retest administrations for any given participant. The two books each include 16 pages with story content. The scripts for the two versions are otherwise identical. Assignment of version to test and retest was randomly determined across participants, with 50 of the 106 participants receiving book A at test.

Manuals for conversation and narration are available at https://ctscassist.ucdmc.ucdavis.edu/ctscassist/surveys/?s=W9W99JLMNX. Included are procedures for administration, training, and assessment of fidelity.

### ELS examiner training and fidelity

Prior to testing any participants, each examiner was trained to ensure fidelity of administration of the conversation and narration procedures. Training began with a team from the lead site that developed the ELS procedures visiting each of the other four sites to discuss the protocol and answer questions about the procedures. Thereafter, each examiner then reviewed written instructional manuals and viewed video recordings of administrations selected from a library of previous “gold standard” administrations. Videos spanned the age range of interest here and included individuals with FXS and other neurodevelopmental disorders, as well as TD children. Video teleconferences, phone calls, and emails were then used as needed to answer questions from the examiners at the collaborating sites. Each examiner then practiced administration, first with another typical adult, before administering to a TD child and then an individual with a developmental disability. A scoring rubric was used to evaluate each practice administration, with a score of 90% correct on critical administration components required to be at fidelity to complete training. Each examiner had to be reliable on one TD child and one individual with an ID before being cleared to test participants. Written feedback and the scoring rubric were shared with the examiner after each administration. After training, fidelity was assessed on 13 randomly selected administrations of conversation and narration with and between sites. The mean (and range of fidelity scores) was 94% (81–100%). Of the samples reviewed for fidelity, one narration (81%) and three conversation samples (86%, 88%, and 89%) fell slightly below the 90% threshold established a priori for fidelity.

### Transcription and coding of expressive language samples

All conversational and narrative samples were audio-recorded using digital recorders. These samples were transcribed by highly trained assistants following transcription procedures developed previously, which have been shown to yield adequate levels of inter-transcriber reliability [31, 34, 62]. The transcription process involved a first draft by a primary transcriber, feedback by a secondary transcriber, and final editing by the primary transcriber. The use of Systematic Analysis of Language Transcripts, 2018 Research Version (SALT) [66] guided transcription. SALT is a computer program that allows standard and user-defined analyses of transcripts prepared as text files according to well-established conventions in child language research, although we have added additional conventions and decisions rules over the years based on the unique characteristics of individuals with ID and the contexts in which we sample their language. All transcription was conducted at a single site (UC Davis MIND Institute).

In preparing the transcripts, talk was segmented into Communication-units (C-units), with a C-unit defined as an independent clauses with associated modifiers, including dependent clauses [67]. In practice, non-clausal utterances such as sentence fragments and elliptical responses also constitute C-units, and thus, the independent clause (and modifiers) is actually the upper bound for segmentation [68]. The C-unit provides a more accurate measure of language ability than does segmentation into only utterances for speakers beyond a developmental level of 3 years [31]. Note that in the case of utterances that include an unintelligible portion, the transcriber relies only on unambiguous syntactic cues to make decisions about segmentation [31, 32, 34]. For example, “I went to the store and I bought XX” (where "XX denotes unintelligible speech) would be segmented into separate C-units at the word “and,” which conjoins independent clauses; however, the transcriber would not segment the unintelligible portion any further regardless of its length.

Transcribers were required to achieve agreement with a gold standard transcription, with different a priori levels established for different dimensions of the transcription process (e.g., segmentation to C-units, number of morphemes). Transcribers were blind to (1) diagnosis (i.e., FXS, Down syndrome, or ASD), (2) whether the sample was from test or retest, and (3) results of other measures completed by the participant. In addition, the transcribers did not work exclusively on samples for participants with FXS; instead, also typically conducting transcription of samples from other diagnostic groups and other studies in parallel with the samples of interest in this study.

We evaluated inter-transcriber agreement by making comparisons between transcripts prepared independently by two different teams (i.e., a different primary and secondary transcriber). We randomly selected six compliant conversations and six compliant narrations. This set included at least three samples for each of the three age groups and at least two per data collection site. Only dimensions of transcription relevant to the dependent measures of this study were examined. Inter-transcriber agreement averaged 89% across the relevant dimensions of transcription: 84% for segmentation into C-units, 86% for identification of partly or fully unintelligible C-units, 97% for identification of complete C-units, 94% for identification of C-units containing mazes, 86% for identification of the exact number of morphemes in each C-unit, 85% for identification of the exact number of words in each C-unit, and 89% for identification of the exact lexical and morphemic content of each C-unit. For the last three dimensions, we required that the two transcriptions were in complete agreement for a C-unit, which is a conservative approach to agreement. For example, a C-unit represented as eight morphemes in one transcript and as nine in the second transcript would be scored as a disagreement (i.e., 0% agreement rather than as 89% agreement) for the number of morphemes dimension, and a C-unit transcribed as “the boy patted a dog” in one transcript and as “the boy patted the dog” in the second transcript would be scored as a disagreement (i.e., 0% agreement rather than as 80% agreement) for the lexical and morphemic content dimension. These agreement percentages are similar to those in previous studies of typical and atypical samples (e.g., [31, 34, 62]). We also used intraclass correlations to capture the similarity between the two transcriptions of each of the 12 samples in terms of the five primary outcome variables of interest (described below). The intraclass correlations ranged from .84 to .99 with *p* < .005 in all cases, indicating strong agreement between transcriptions.

### Measures derived from expressive language samples

We computed five primary measures for each language sample in the present study, with all of them computed automatically by SALT or with minimal transformation of SALT-generated variables. Examining these measures separately, rather than creating a single composite, is likely to be important for clinical trials, which may involve pharmacological agents hypothesized to have highly circumscribed effects on language (e.g., increasing talkativeness and language complexity by reducing social anxiety) which might be masked by a composite. The utility of these particular measures in tracking developmental change and discriminating typically and atypically developing individuals has been established [31, 34, 55, 56, 62]. Additional measures, which require more extensive “hand” coding, such as verbal preservation and pragmatic appropriateness, are also being explored and will be reported in subsequent papers.

#### Talkativeness

Number of C-units attempted per minute. This measure provides an estimate of the motivation to talk.

#### Unintelligibility

Proportion of the total C-units that are marked as either partly or fully unintelligible by the transcriber. This measure indexes problems in speech articulation.

#### Dysfluency

Proportion of the total number of complete and fully intelligible C-units that include a maze or verbal dysfluency (e.g., um, uh, er, or a partial repetition of a word). This measure indexes problems in language planning.

#### Lexical diversity

Number of different word roots in 50 complete and fully intelligible C-units (or the full sample if less 50 C-units were produced). This measure reflects the size of the participant’s expressive vocabulary.

#### Syntax

Mean number of morphemes per C-unit (MLU) in 50 complete and fully intelligible C-units (or the full sample if less than 50 C-units), which is an omnibus measure of syntactic maturity.

### Construct validity measures

We administered a battery of standardized tests and informant reports chosen to capture all the dimensions of language ability and performance assessed with the ELS procedures. We hypothesized that these measures would correlate significantly with the ELS-derived measures of the corresponding dimensions of language. For any given participant, different examiners administered the construct validity measures and the ELS procedures. Moreover, ELS results for any given participant were not shared with the examiner administering the construct validity measures to the participant. There were missing data values for some participants on some measures because of scheduling difficulties, participant noncompliance, and examiner error.

The *Clinical Evaluation of Language Fundamentals-4th edition* (CELF-4) [69] is a comprehensive, individually administered standardized test designed for ages 5 through 21 years. We administered the CELF-4 *Formulated Sentences* (FS) subtest, which measures syntax, and thus, was expected to correlate with the syntax measure in the language samples. We also administered the *Expressive Vocabulary* (EV) subtest, which was expected to correlate with the ELS lexical diversity measure. To minimize floor effects, we used raw scores (i.e., number correct) rather than standard scores for each CELF-4 subtest, with all participants starting at item 1 and continuing until the ceiling criteria were reached.

The *Verbal Working Memory* subtest of the *Stanford-Binet-5th edition* (SB5 VWM) was administered and expected to correlate with the ELS dysfluency measure (i.e., proportion dysfluent C-units). This subtest requires storing and manipulating verbal information and planning a verbal response, with the specific processes engaged varying across items. The least difficult items require the immediate and exact repetition of phrases and sentences, whereas the more difficult items require recalling the last words of questions that have been previously answered. We used raw scores from the SB5 VWM subtest.

It should be noted that we had originally planned to use the Rapid Automatic Naming subtest of the CELF-4 as the validation measure for ELS dysfluency; however, the measure proved too difficult for many of the participants and even several simplifying adaptations were not adequate to yield meaningful data. Nevertheless, we found that for the 31 participants with FXS who had valid Rapid Auditory Naming data, their scores correlated significantly with their SB5 VWM scores (*r* = .423, *p* = .020) and the relationship with dysfluency was the same.

The *Goldman-Fristoe Test of Articulation* (GFTA) [70] provided a measure of speech articulation and potential correlate for the unintelligibility measure derived from the ELS (i.e., proportion unintelligible C-units). The GFTA is designed for ages 2 through 21 years. In the present project, articulation accuracy was scored from imitative single-word production. We used the percentage of correct phonemes produced in single words (SiW) in all analyses. All samples were audio-recorded using digital recorders and were scored at UC Davis.

The *Vineland Adaptive Behavior Scales, Second Edition* [59] was completed as a self-administered questionnaire by the parent/guardian. The VABS-II was normed for ages 3 to 21 years. We used the raw score from the Expressive Communication (EC) domain. We expected that this score would reflect a combination of communication skill mastery and the motivation to engage in communication; thus, we expected it to correlate with ELS talkativeness (i.e., the number of C-units attempted per minute).

### Statistical analysis

We conducted parametric analyses to examine the psychometric properties of the ELS-derived measures: *t* tests to evaluate practice effects, Pearson correlations and intraclass correlations to evaluate test-retest reliability, and Pearson correlations to evaluate convergent and discriminative construct validity. In each of these parametric analyses, we corrected for conducting multiple tests using the false discovery rate (FDR) procedure of Benjamini and Hochberg [71] to maintain a familywise alpha rate of *p* < .050; however, we also present the uncorrected *p* values to provide additional information to eventual users of these outcome measures. In applying the FDR, we corrected for familywise error rate with a family defined by participant group and sampling context; for example, in the primary analyses involving the full sample of participants, the tests for the conversational measures formed one family and those for the narrative measures a second family, and in the exploratory analyses by age group, each age group X sampling context combination constituted a different family of tests.

Note that because several variables were not normally distributed (e.g., the unintelligibility measure was negatively skewed), we also conducted nonparametric analyses where appropriate nonparametric alternatives existed. The parametric and nonparametric analyses yielded similar results for the most part. In a few instances, however, an individual parametric test that was significant at *p* < .05 failed to reach significance in the nonparametric equivalent and vice versa. We have noted these instances in the accompanying tables, but take the parametric analyses as primary in terms of conclusions.

## Results

### Feasibility

We computed several measures to determine the extent to which the participants were able to complete the conversational and narrative procedures in meaningful ways. These data are presented in Table 2. Note that these measures overlap despite taking a somewhat different approach to indexing meaningful task engagement.

**Table 2:** Feasibility of ELS procedures: compliance, number of C-units per sample, and completeness

ELS procedure	Test	Retest
**Conversation**		
Noncompliant	15 (14.2%)	6 (5.7%)
< 50 C-units (total)	5 (4.7%)	4 (3.9%)
< 50 C-units (analysis set)	11 (10.4%)	12 (11.7%)
Incomplete conversations^a^	16 (15%)	11 (11%)
**Narration**		
Noncompliant	15 (14.2%)	9 (8.5%)
< 25 C-units (total)	13 (12.3%)	10 (9.7%)
< 25 C-units (analysis set)	27 (25.5%)	22 (21.4%)
Incomplete narratives^b^	27 (25%)^c^	22 (21%)^d^

Cell values are number (and percentages) of participants, with *n* = 106 for first and 103 for retest administrations, respectively, unless noted otherwise

^a^Defined in terms of duration

^b^Defined in terms of coverage of pages

^c^*n* = 105

^d^*n* = 102

First, examiners and transcribers were instructed to notate whether the participant was noncompliant, and we computed the rate of noncompliance they noted, with noncompliance defined as explicit refusal to do the task (e.g., saying, “I’m done”) or repeated off-task behavior. A relatively small percentage of participants were judged to be noncompliant in either conversation or narration, with somewhat less noncompliance on retest than the on the initial administration.

Second, we considered the number of C-units produced in each procedure as an indication of meaningful task engagement. We adopted the criterion of 50 (or more) C-units in conversation and 25 (or more) C-units in narration as indicators of meaningful engagement. These values seemed to be reasonable based on (a) the demands of our ELS procedures (e.g., producing at least 5 utterances per minute in conversation and at least one descriptive utterance for each of the 16 pages in the books for narration) and (b) our previous research on the ELS procedures with a number of populations functioning at roughly the developmental levels represented in the present sample of participants [31, 34, 62, 64]. We examined both the total number of C-units and the number of complete and intelligible C-units relative to these values. The former is used in calculating our talkativeness and unintelligibility measures, whereas the latter is used in calculating the dysfluency, syntax, and vocabulary measures. Noncompliant participants were included in these analyses. As can be seen in Table 2, the vast majority of participants met these C-unit thresholds in conversation. In narration, only three quarters met the threshold for the analysis set, although better than 90% met the threshold for the total number of C-units. It is important to note that these C-unit targets are hypothesized indicators of engagement. It is not yet clear that these values must be met for a valid measure of an individual’s language skills.

Third, we examined the number of participants who produced expressive samples that were “complete.” In the case of conversation, which was a timed task with a target of 10 min, we considered a sample complete if the sample was at least 9.5 min in duration, which allowed for some minimal variation due to examiner error or scheduling challenges. In the case of narration, complete was defined as the production of at least one task-relevant C-unit for each of the 16 pages in the book. Noncompliant participants were included in these analyses. As seen in Table 2, the participants were, as a group, largely successful in producing complete conversations and, to a somewhat lesser extent, complete narrations.

We also compared the characteristics of compliant and noncompliant participants (see Table 3). In terms of conversation, the participants who were compliant on both the first and retest administrations differed significantly (according to the FDR correction) from those who were noncompliant on one or both in terms of CA, the Sansone et al. [23] SB5 deviation scores and the ADOS-2 overall severity score, with greater compliance associated with older age, higher IQ, and less severe ASD symptoms. In terms of narration, the participants who were compliant on both administrations also differed significantly from those who were noncompliant on one or both in terms of CA, the Sansone et al. SB5 deviation scores, and the ADOS-2 overall severity score, again, in the expected direction.

**Table 3:**
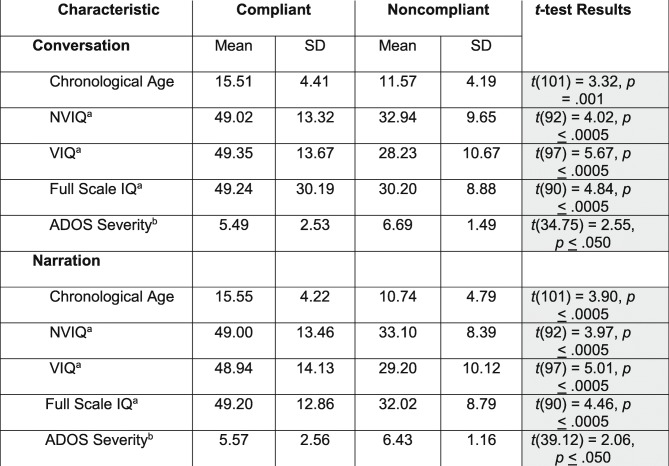
Characteristics of compliant and noncompliant participants

Shaded cells reflect *p* values that are significant according to the FDR procedure. *n* for compliant ranges from 81 to 87 per measure for conversation and 83 to 89 for narration. *n* for noncompliant ranges from 15 to 16 per measure for conversation and 13 to 14 for narration

^a^Stanford-Binet, 5th edition transformed scores (Sansone et al. [23])

^b^Total severity score from Autism Diagnostic Observation Schedule, 2nd edition

Comparisons were also conducted on the characteristics of the groups defined by verbal output. In comparing participants who produced 50 or more C-units in both the first and retest conversation administrations to participants who did not achieve this level of output, no significant differences emerged in terms of age, IQ, or ADOS-2 severity scores. Similarly, there were no significant differences between the participants who produced 25 or more C-units in both the first and retest narration administrations to participants who were less verbally productive. In contrast, significant differences (with the FDR correction) emerged between participants in terms of the subgroups defined by the rate of complete and intelligible C-unit production. In conversation, a significant difference was found for the SB5 verbal scale composite deviation score (*t*[103] = 2.90, *p* = .005), with greater verbal output associated with higher SB5 verbal scores. In terms of narration, significant differences between the groups were found for the SB5 nonverbal (*t*[75.87] = 3.89, *p* < .0005), verbal (*t*[103] = 3.35, *p* < .0005), and full-scale (*t*[70.72] = 3.59, *p* = .001) deviation score composites, with greater verbal output associated with higher SB5 scores.

In the analyses reported in the remainder of this paper, we excluded participants who were noncompliant according to examiner or transcriber ratings, but we did not exclude participants who generated incomplete samples or samples under the target sizes in terms of number of C-units. We felt it important from the perspective of future treatment studies to understand the psychometric properties of the ELS measures for the least restricted sample as possible; thus, we felt only obviously noncompliant participants should be excluded because even a small amount of talk may be a useful indicator of language capacity and change.

### Practice effects and test-retest reliability

The data on the relationships between the two administrations of the ELS procedures are presented in Tables 4 (practice effects) and 5 (test-retest reliability). We included in these analyses only those participants who were compliant on both administrations of the ELS procedure of interest regardless of the number of C-units a participant produced. As can be seen in Table 4, the means for each of the measures change relatively little from the first to the second administration in either conversation or narration, suggesting the absence of practice effects. In fact, *t* tests for paired samples indicated that the difference in means from test to retest did not reach a *p* < .050 level of significance for any of the 10 tests even before application of the FDR.

**Table 4:** Practice effects on repeated administrations over a 4-week interval

	Conversation (***n*** = 87)				Narration (***n*** = 88)			
Measure	Visit 1		Retest		Visit 1		Retest	
	***M***	SD	***M***	SD	***M***	SD	***M***	SD
**Lexical diversity**	77.99	31.78	82.43	34.01	67.13	31.66	69.56	31.97
**Syntax**	3.64	1.39	3.81	1.46	4.80	2.13	4.96	2.20
**Talkativeness**	14.56	5.40	14.84	4.96	12.24	5.59	12.15	5.11
**Unintelligibility**	0.15	0.13	0.16	0.15	0.16	0.17	0.16	0.16
**Dysfluency**	0.20	0.13	0.21	0.13	0.17	0.12	0.19	0.14

None of the *t* tests comparing the first and retest administrations was significant at *p* < .050 (even uncorrected for multiple tests)

As can be seen in Table 5, there was excellent consistency from the first to the retest administration in the participants’ scores on each of the measures in both conversation and narration. We computed both simple bivariate Pearson correlations as well as intraclass correlations between the first and second administration for each of the five measures in conversation and in narration. In computing the intraclass correlations, we report results for a mixed model, assuming no interaction, and absolute agreement. All bivariate and intraclass correlations were significant, even after use of the FDR procedure.

**Table 5:**
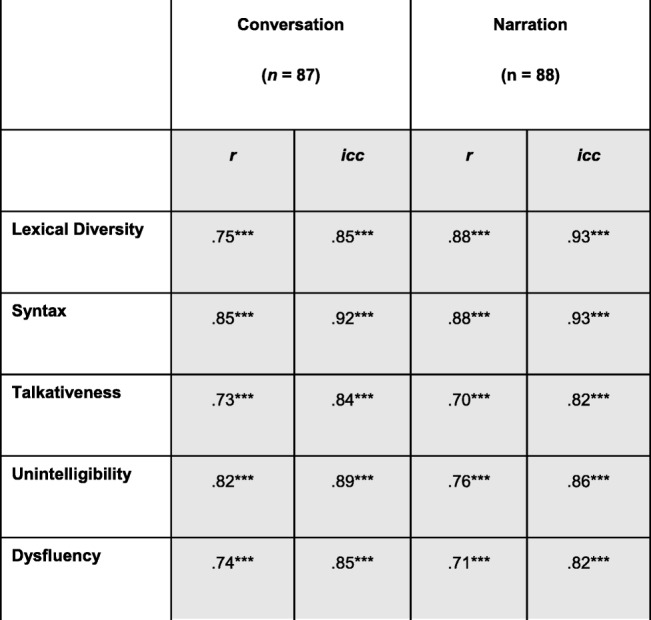
Test-retest reliability over a 4-week interval: bivariate correlations and intraclass correlations

Note that uncorrected *p* values for individual tests are marked with asterisks as follows: ***signifies *p* ≤ .0005; **signifies *p* ≤ .005; **signifies *p* ≤ .050. Shaded cells contain values that were significant at *p* ≤ .050 after correcting for multiple tests through the FDR procedure

### Construct validity

Data on construct validity are presented in Tables 6 and 7 for conversation and narration, respectively. Only participants who were compliant on both administrations of conversation (*n* = 87) are included in the analyses presented in Table 6, and only those compliant on both administrations of the narrative (*n* = 89) are included in Table 7. The analyses were conducted, however, only for the data from the first administration of the ELS procedures because the external validation measures were administered only then and not at the 4-week retest. We examined the relationships among variables by computing zero-order bivariate correlations, with the exception of the relationship between dysfluency and its validation measure. In the case of dysfluency, there is considerable evidence that the variable is positively correlated with syntactic complexity [72]. Indeed, multiword speech would seem to be a prerequisite for filled pauses and other forms of dysfluency. In analyzing the relationship between the dysfluency measure and the SB5 VWM measure, therefore, we controlled for MLU, and this partial correlation is reported in Tables 6 and 7.

**Table 6:**
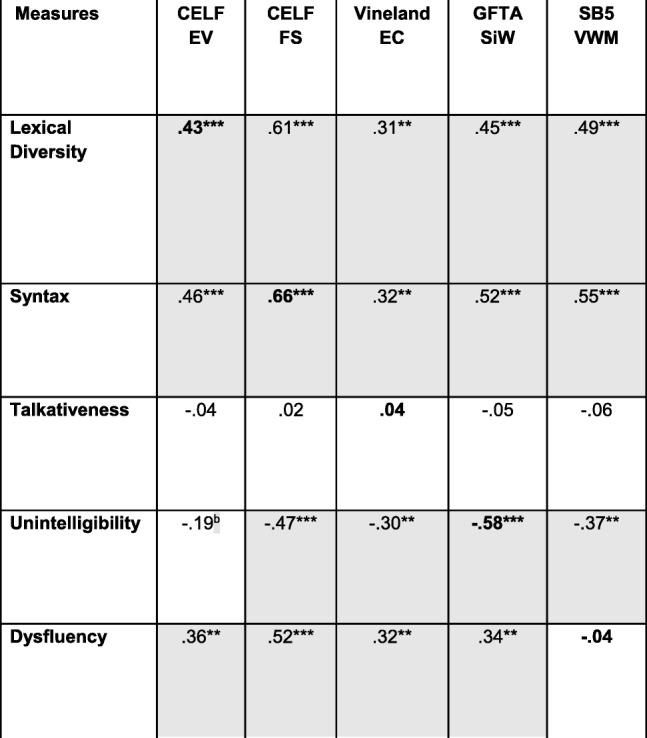
Construct validity: conversation

Note that all values are bivariate zero-order correlations except for that between dysfluency and the SB5 VWM score, which is a partial correlation controlling for syntax (MLU). *n* = 83–87 across correlations. Uncorrected *p* values for individual tests are marked with asterisks as follows: ***signifies *p* ≤ .0005; **signifies *p* ≤ .005; **signifies *p* ≤ .050. Shaded cells contain values that were significant at *p* ≤ .050 after correcting for multiple tests through the FDR procedure. The boldfaced values represent convergent validity relationships; all other values represent discriminant validity relationships

^a^*p* > .050 in Spearman (nonparametric) analysis

^b^*p* ≤ .050 in Spearman (nonparametric) analysis

**Table 7:**
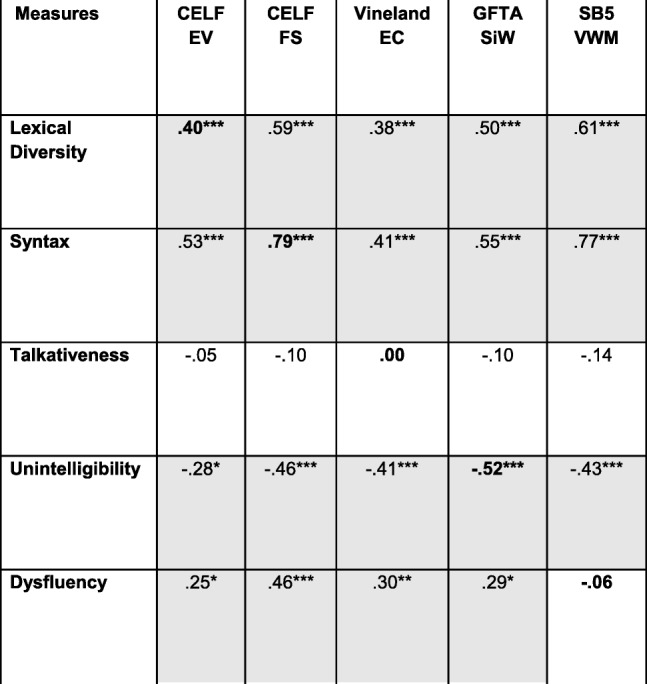
Construct validity: narration

Note that all values are bivariate zero-order correlations except for that between dysfluency and the SB5 VWM score, which is a partial correlation controlling for syntax (MLU). *n* = 85–89 across correlations. Uncorrected *p* values for individual tests are marked with asterisks as follows: ***signifies *p* ≤ .0005; **signifies *p* ≤ .005; **signifies *p* ≤ .050. Shaded cells contain values that were significant at *p* ≤ .050 after correcting for multiple tests through the FDR procedure. The boldfaced values represent convergent validity relationships; all other values represent discriminant validity relationships

The diagonals in the tables (boldface type) contain the correlations between the ELS-derived measures and the external validation measures administered to establish convergent validity. The results were consistent for conversation and narration. In particular, the correlations with the external validation measures were significant (after the FDR correction) for the lexical diversity, syntactic, and unintelligibility measures for both conversation and narration, suggesting strong convergent validity. These correlations are in the range of what Cohen [73] would describe as medium to large correlations in the behavioral sciences. The corresponding correlations for talkativeness and dysfluencies were nonsignificant and essentially near zero.

The off-diagonal cells in Tables 6 and 7 present the correlations that are relevant to evaluating the discriminant validity of the ELS-derived measures. As seen in the tables, most of the standardized tests used to establish construct validity generally correlated significantly with each of the ELS-derived measures of lexical diversity, syntax, unintelligibility, and dysfluency. In addition, with the exception of talkativeness, the ELS-derived measures were highly intercorrelated (Tables 8 and 9). In general, a similar pattern of intercorrelations was observed in the subsequent analyses involving stratification of the sample by age, IQ, and ASD symptom severity, although the small sample size for the youngest age group led to fewer correlations reaching significance. Thus, there is a lack of specificity, or discriminant validity, in the both the ELS-derived measures and the standardized tests.

**Table 8:**
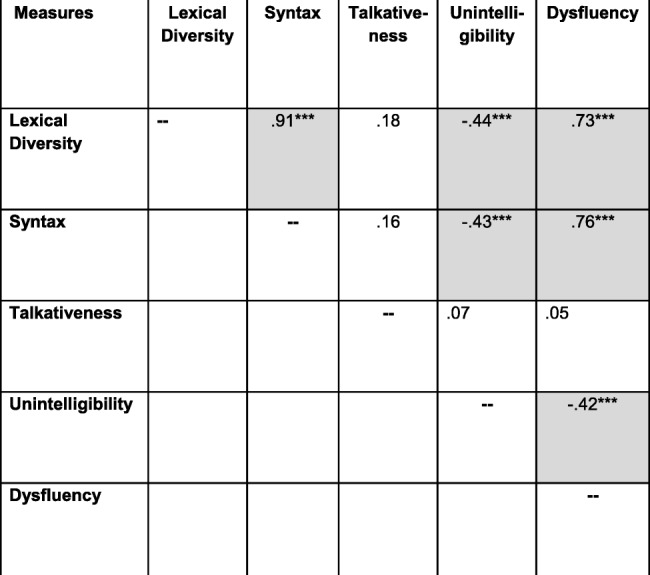
Intercorrelations among ELS measures: conversation

Note that all values are bivariate zero-order correlations. *n* = 87 for all correlations. Uncorrected *p* values for individual tests are marked with asterisks as follows: ***signifies *p* ≤ .0005; **signifies *p* ≤ .005; **signifies *p* ≤ .050. Shaded cells contain values that were significant at *p* ≤ .050 after correcting for multiple tests through the FDR procedure

**Table 9:**
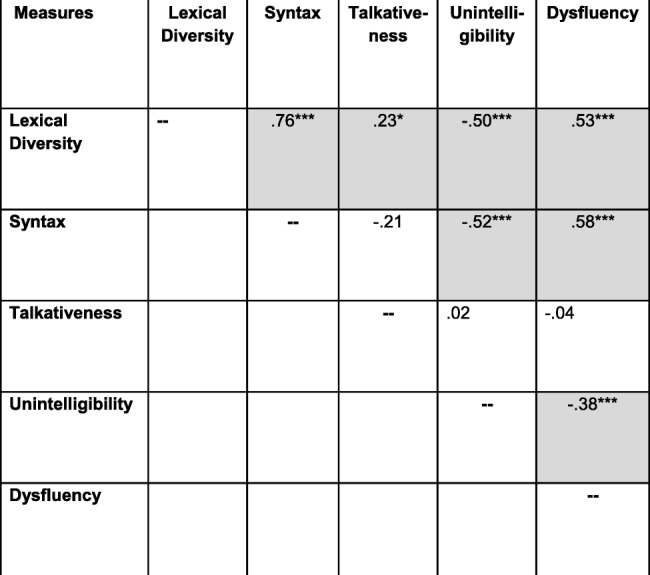
Intercorrelations among ELS measures: narration

Note that all values are bivariate zero-order correlations. *n* = 89 for all correlations. Uncorrected *p* values for individual tests are marked with asterisks as follows: ***signifies *p* ≤ .0005; ** signifies *p* ≤ .005; ** signifies *p* ≤ .050. Shaded cells contain values that were significant at *p* ≤ .050 after correcting for multiple tests through the FDR procedure

### Psychometrics of ELS measures by age group

We examined practice effects, test-retest reliability, and construct validity of the ELS measures separately for each of the three age groups. These analyses were limited, however, by the fact that despite our intent to recruit equal numbers of participants in each age group, we had the most success recruiting for the 12- to 17-year-old group. In addition, the analysis of compliance presented for the full sample suggested that younger participants were most likely to be judged as noncompliant by the examiners and transcribers. Indeed, examining noncompliance as a function of age group further clarifies this latter finding. In particular, 11 of the 28 (39%) 6- to 11-year-olds were noncompliant on at least one of the two conversation administrations compared to 5 of 55 (9%) and 1 of 23 (5%) of the 12- to 17-year-olds and 18- to 23-year-olds, respectively. The corresponding figures for narration were 10 (36%), 8 (15%), and 1 (5%). Thus, statistical power was more limited for the youngest age group and the findings for this group should be interpreted cautiously. Nevertheless, the analyses uncovered important differences and similarities in how the ELS procedures function across the three age groups. The analyses reported in the remainder of this section include only those participants who were compliant on both administrations of the ELS procedure of interest.

In terms of practice effects, we compared the mean scores on each of the five ELS-derived measures on the first and retest administrations for those participants who were compliant on both administrations (see Supplementary Table 1). There were no significant differences on any measure for the 6- to 11-year-old group on either conversation or narration. Although the sample sizes were small for this youngest group (18 for conversation and 17 for narration), it is worth noting that none of the 10 *t* test comparisons of the first and retest scores approached significance, with (uncorrected) *p* values ranging from .18 to .71 for conversation and .15 to .94 for narration. For each of the other two age groups, none of the *t* tests reached significance according to the FDR correction.

We again examined test-retest reliability by computing Pearson correlations and intraclass correlations for each age group separately (see Supplementary Table 4). For each of the two oldest age groups, the Pearson and intraclass correlations between the first and retest administrations were significant after the FDR correction for all five ELS-derived measures for conversation and for narration, thereby replicating the findings for the combined sample. In the case of the 6- to 11-year-old group, however, the test-retest findings were less consistent. In particular, for this youngest age group, the test-retest correlations were generally of lesser absolute magnitude relative to the two older groups. Nonetheless, either the bivariate or the intraclass correlation was significant after FDR correction for every measure in at least one context for this youngest group.

We examined construct validity by again computing correlations between the ELS-derived measures and the various standardized tests as we did in the analyses for the full sample (see Supplementary Table 7 and 8). For the youngest age group, none of the correlations addressing convergent validity was significant after the FDR correction. The construct validity findings for the 12- to 17-year-old groups were the same as for the full sample; that is, significant correlations after the FDR correction between the ELS syntactic, lexical, and unintelligibility measures and their corresponding validation measures for conversation and for narration. For the oldest age group, we found significant correlations between the validation measures and the ELS syntactic and unintelligibility measures for conversation and for narration; however, the correlations between lexical diversity and the CELF4 EV subtest were not significant for conversation or narration. As in the full sample, there were also significant correlations between the ELS syntactic and unintelligibility measures and several of the standardized tests, suggesting weak discriminant validity.

### Psychometrics of ELS measures by IQ group

We examined practice effects, test-retest reliability, and construct validity of the ELS measures as a function of participant IQ. We did this by assigning the participants into a lower and higher IQ group via a median split using the extended SB5 full scale extended deviation IQs (Sansone et al. [23]). We did this separately for the analyses focused on conversation and narration and included only the participants who were compliant on both the first and retest administrations of the relevant sampling conditions. The median IQ was 47.62 (*n* = 81) for the conversation analyses and 47.73 (*n* = 80) for the narration analyses.

We examined practice effects by conducting *t* tests for correlated samples comparing the means on the first and retest administrations for each of the ELS-derived conversation and narration measures for those participants who were compliant on both administrations (see Supplementary Table 2). There were no significant differences on any measure in conversation or narration for either the lower IQ or higher IQ group. These results replicate those from the analyses of the full sample of participants.

We examined test-retest reliability by computing correlations and intraclass correlations between the two ELS administrations for each IQ group separately. For both IQ groups, the Pearson correlations and the intraclass correlations between the first and retest administrations for all conversation and narration measures were significant, even after application of the FDR correction (see Supplementary Table 5), thereby replicating the results for the full sample of participants.

Construct validity was examined by computing correlations between the ELS measures and the various standardized tests as we did in the analyses for the full sample of participants (see Supplementary Table 9 and 10). For both IQ groups, the correlations between the ELS syntactic measure and the CELF-4 FS subtest were significant in conversation and narration. The correlation between the ELS measure of unintelligibility and the GFTA score was significant in narration, but only approached significance after FDR correction in conversation. In contrast to the results for the analyses of the full sample of participants, lexical diversity was not correlated significantly with the CELF4 EV subtest in either conversation or narration for either IQ group. In general, the absolute magnitude of the convergent validity correlations was greater in the higher IQ group. As in the full sample, there was not strong discriminant validity for the ELS measures.

### Psychometrics of ELS measures by autism status

We examined practice effects, test-retest reliability, and construct validity of the ELS measures as a function of participant ADOS-2 severity score. We did this by creating two groups: a low severity group, which was comprised of participants who received a score of 4 or less (i.e., ADOS-2 Comparison scores classifications reflecting minimal to no evidence or low levels of ASD symptomatology), and a high severity group, which included participants who received a score of 5 or higher (i.e., ADOS-2 Comparison scores classifications reflecting either moderate or high levels of ASD symptomatology). In the analysis of conversational samples, we included only participants who were compliant on both administrations, and this resulted in 26 participants in the low severity group and 55 in the high severity group. The corresponding sample sizes for the narrative samples were 27 and 56. Although this binary ASD classification resulted in unequal sample sizes and more limited statistical power for the analyses for the low severity group, we felt that the classification was clinically meaningful.

Practice effects were again evaluated by conducting *t* tests for correlated samples to compare the means on the first and retest administrations for each conversation and narration measure for those participants who were compliant on both administrations (see Supplementary Table 3). In conversation, there were no significant differences between the two administrations for either severity group, even before application of the FDR correction. In narration, there was a significant difference between the two administrations for dysfluency for the low severity group, with dysfluencies actually becoming proportionally more frequent in retest (.30) relative to the first administration (.23). None of the other tests of the difference between the first and retest administrations approached significance at *p* < .050.

We examined test-retest reliability by computing Pearson correlations and intraclass correlations between the two ELS administrations for each ASD severity group separately (see Supplementary Table 6). For both ASD severity groups, the Pearson and intraclass correlations between the first and retest administrations were all significant after the FDR procedure for every ELS measure in both conversation and narration. These analyses replicate those for the full sample of participants.

We examined construct validity as in the analyses for the full sample of participants (see Supplementary Table 11 and 12). For the low ASD severity group, the correlation between the ELS syntactic measure and the CELF4 FS subtest was significant for both conversation and narration. ELS unintelligibility in conversation also correlated significantly with its respective validation measure for the low severity group. The correlation between lexical diversity and the CELF-4 EV subtest approached significance in narration for the low ASD severity group. For the high ASD severity group, the ELS syntactic, lexical, and unintelligibility measures were each correlated significantly with their respective validation measure in conversation and in narration after the FDR correction, consistent with findings for the full sample of participants. As in the full sample, the ELS lexical, syntactic, and unintelligibility measures also tended to correlate with multiple standardized measures, suggesting a lack of discriminant validity.

## Discussion

The goal of the present study was to evaluate the psychometric properties of five measures, collectively indexing a range of expressive language skills, derived from two ELS procedures (conversation and narration) designed to be consistently administered across participants and occasions and with minimal influence of the examiner on the participant’s language output.

### Psychometric findings for the full sample

We found that for the sample of 6- to 23-year-olds with FXS as whole, the vast majority of participants were compliant with, and meaningfully engaged by, the conversation and narration procedures. Noncompliance rates for both ELS procedures were under 15%. In addition, fewer than 15% of the participants fell under the thresholds for duration or number of C-units produced for conversation. The narration procedure was more challenging for the participants, however. One quarter of the participants fell below the threshold for narrative completeness or below the minimum targets for number of complete and intelligible C-units produced. Note, however, that a narrative sample that is incomplete or falls short of the threshold in terms of number of C-units does not necessarily preclude analysis nor is it necessarily a poor reflection of a participant’s expressive language skill, as evidenced by the generally strong psychometric properties we observed for the ELS narration measures for compliant participants. This finding suggests that even very brief samples, which place minimal testing burden on the participants and lessen the resources needed for transcription, can yield valid data for individuals with FXS in the context of a clinical trial.

The greater challenge posed by narration may reflect the fact that, unlike conversation, narration required sustained attention to a task (i.e., the book) and an orderly page-by-page progression through the task according to a pace set largely by the examiner. We do not suggest, however, including only conversation as the source of ELS outcome measures in a treatment study. The vast majority of the participants were still meaningfully engaged and compliant in narration. Moreover, it has been documented in several previous studies [31, 34] that narration and conversation “pull” for different language skills, and thus, when used together, they are likely to provide a more comprehensive characterization of an individual’s language capabilities than either alone.

We also found that noncompliance was greatest for younger and more severely affected individuals (i.e., lower IQ and more severe ASD symptoms). Interestingly, there is evidence from other treatment studies and measure evaluation studies involving individuals with intellectual disabilities that many widely used measures also have high “failure rates” in this range. In a measure evaluation study conducted by Edgin et al. [74], for example, approximately one-third of 7- to 20-year-olds with Down syndrome were at the floor on the CANTAB Spatial Span subtest because of either noncompliance or an inability to achieve a sufficient number of correct responses. Similarly, Hessl et al. [22] administered the NIH Toolbox Cognitive Battery, which was normed on children with TD as young as 3 years, to 6- to 25-year-olds with ID. Hessl et al. found that between 28 and 53% of the participants did not provide meaningful data for three of the seven subtests. The development of adequate measures for younger individuals with ID is particularly challenging, and more research should be conducted to identify or develop new methods of assessing these groups. In the interim, results for the ELS procedures examined in this study should be interpreted cautiously when incorporated into treatment studies with young school-age children with FXS or ID more generally.

It is important, although not straightforward, to compare noncompliance rates for the ELS procedures to that of the standardized language tests. On the one hand, every participant in the present sample was able to achieve a score on the CELF-4 subtests we administered as well as on the GFTA, suggesting a high level of compliance with the demands of those measures. On the other hand, the discrete, item-by-item, nature of those measures may allow a response, but not always with meaningful engagement on the part of the participant. For example, consider the participants who were noncompliant in conversation (according to the examiner or transcriber). Of those participants, 50% did not achieve a single correct response on the FS subtest of the CELF-4, and 75% achieved no more than a single correct response on that subtest. For the participants who were judged as noncompliant in narration, the corresponding values for the FS subtest were 57% and 86%. Additionally, approximately 80% of participants judged fully compliant in conversation or narration achieved a score of 2 or more correct on the CELF-4 ES subtest. These findings suggest that the rates of meaningful engagement for individuals with FXS in the age and ability range studied may not be too dissimilar for the ELS procedures and at least some standardized tests.

There were minimal to no practice effects evident for any of the five ELS measures in either conversation or narration for compliant participants. Practice effects are particularly problematic in open-label clinical trials and single-participant design treatment studies, making it virtually impossible to conclude that observed changes over the course of a study are actually signs of treatment efficacy. Even in randomized-controlled trials, practice effects on an outcome measure are problematic, especially if the practice leads to improvements near the ceiling of the measure. In such cases, it becomes difficult for the treatment to have an effect over and above that of sheer practice. The lack of meaningful practice effects for the ELS measures makes them well suited to treatment studies, particularly given that most such studies involve longer intervals between measure administrations than the 4-week window of the present project.

We found evidence of strong test-retest reliability for all ELS measures in both conversation and narration. More concretely, these data demonstrate that there is consistency at the level of the individual in his or her performance from one administration to the next, at least over a 4-week interval. Such consistency is not guaranteed by a lack of practice effects at the level of the group. It is possible for there to be no changes in group mean scores from the first to the retest administration, but still be dramatic changes among individuals from one administration to the next. Our data indicate that individuals largely maintained their relative rankings and relative “distances” from each other on each ELS measure across the two administrations. This “guarantee” that a participant’s score on the outcome measure will not change in the absence of a treatment or an intervention not typical of daily life is of critical importance to the design of any treatment study, whether pharmacological or behavioral/educational. It should be noted, however, that at this point we can only address issues of reproducibility over the relatively short interval of 4 weeks, an interval we selected to minimize the possibility of developmental change in expressive language skills among individuals with FXS. The reliability of the ELS measures over longer intervals, thus, remains to be determined.

We examined the construct validity of the ELS measures by examining their correlations with directly administered standardized tests and informant report measures of similar constructs. In terms of convergent validity, the results were consistent across conversation and narration in supporting the convergent validity of the ELS measures of syntax, lexical diversity, and unintelligibility. These ELS measures were significantly correlated with the standardized tests designed to measure similar constructs, thereby further supporting their promise as clinical endpoints in treatment studies. Moreover, these convergent validity correlations were in the medium to large range according to common conventions in behavioral science, which is especially impressive given the very different assessment formats of the ELS procedures and the standardized measures used for validation. Interestingly, a recent study in which the ELS narration procedure was administered to TD children and adults [62] provides additional evidence of construct validity for the syntax and lexical diversity measures. In particular, Channell et al. found that scores on these measures were strongly related to CA, improving linearly from the early school years until reaching a plateau near the end of adolescence. These findings suggest that the measures were useful indicators of language development in the typical population. Although not age-related in the Channell et al. study, we have found in other studies that ELS unintelligibility discriminates between adolescents with FXS and those with Down syndrome [64], with the latter being proportionally more unintelligible, as would be excepted based on the accumulated research with the two disorders [75]. Finally, additional evidence of construct validity is provided by a recent study documenting significant differences on all five ELS measures derived from narration between 5- to 36-year-olds with FXS and CA-matched typically developing individuals [76].

In contrast to the findings for ELS-derived syntax, lexical diversity, and unintelligibility, the correlations of ELS dysfluency and talkativeness with their convergent validity measures were not significant in the present study. This lack of correlation may reflect limitations of the ELS talkativeness and dysfluency measures. In the case of talkativeness, we used number of C-units attempted but ignored the length of those C-units in operationalizing the construct. We did this so as not to “penalize” those participants who had a limited ability to combine words, but yet were as motivated to talk as their more syntactically sophisticated peers. An alternative would be to use the number of words produced as the indicator of talkativeness, which would distinguish between two equally skilled participants who differed in that one provided only brief C-units due to reticence, whereas the other was more expansive, producing longer C-units. In operationalizing talkativeness, we also did not distinguish between spontaneous and prompted responses in part because the scripting of the examiner’s behaviors in our tasks would be expected to minimize differences across participants in the amount of prompted versus spontaneous C-units. We plan to explore the ramifications of different measures of talkativeness in the future.

In the case of dysfluencies, we did not distinguish between different types of filled pauses, reflecting the fact that, for typically developing individuals, this variable has been found to increase slightly with increases in the speaker’s age and the linguistic complexity of the speaker’s productions [62]. Dysfluency also distinguishes among a number of language-related ID conditions [34]. This variable also distinguishes intellectually typical adults who carry a premutation of the *FMR1* gene from their non-carrier peers [77]. Recently, however, evidence has emerged that “um” and “uh” may actually reflect different types of underlying planning process, with the former serving a pragmatic, listener-oriented function [78]. In the future, we will use principal components analysis and other clustering techniques to determine the best set of dysfluencies to include within a single construct.

The lack of evidence for the construct validity of ELS talkativeness and dysfluency may also reflect limitations in the validation measures we selected. In the future, we plan to explore relationships of the ELS measures with other standardized tests and informant report measures. Until further research is conducted, however, we believe that neither talkativeness nor dysfluency, as operationalized in the present study, should be used as an outcome measure in treatment studies involving individuals with FXS, unless these aspects of expressive language are specifically targeted by the intervention, as in behavioral interventions designed to increase participant engagement in talk (e.g., [79]).

Although there was evidence of convergent validity for a subset of the ELS-derived measures, there was no evidence of discriminant validity. The syntactic, lexical, and unintelligibility measures were significantly correlated with each other (as well as with the dysfluency measure), and each correlated significantly with multiple validation measures. This is not surprising given the interrelatedness of the various domains of language in typical development, and that achievements in one domain often set the stage for further developments in other domains (e.g., [80]). As but one example, TD children are able to begin meaningfully combining words in their expressive output only after achieving an expressive vocabulary near 50 words, suggesting that generalizations about syntax are derived from understanding the meanings and combinatorial potential of lexical items [81].

In one sense then, the present construct validity evidence suggests that the ELS measures of syntax, lexical diversity, and unintelligibly are interchangeable as measures of expressive language and that any could be useful clinical endpoints in treatment studies either alone or in combination (i.e., as a composite score). It will be important, however, to gather data about the sensitivity to change of these three measures before ultimately deciding on the best measure(s) for any particular treatment study. In fact, we are currently collecting data on the sensitivity to change in longitudinal assessments in individuals with ID, and the measures are being used in several treatment studies as well, which together will allow evaluation of sensitivity to change. In addition, decisions about which measure to include as a clinical endpoint in any given treatment study should be informed by hypotheses about the particular brain mechanisms targeted by a treatment and the likely specificity of the treatment effects on language.

### Psychometric findings for subgroups of participants

#### Participant age

We found evidence of variation in the psychometric properties of the ELS measures across the three age groups, with the measures working less well for the 6- to 11-year-old participants. Even for this youngest age group, however, the ELS measures appear to hold promise.

In terms of the repeated administrations of the ELS procedures, the data suggest that the measures work reasonably well in terms or reproducibility for all three age groups. There were minimal practice effects, with only a nonsignificant trend (after correction for multiple tests) for an increase in dysfluency from the first to the retest administration in narration for the two older age groups. Interestingly, the increase in dysfluency suggests a decrement in performance, perhaps reflecting increased frustration with the repeated tasks or, alternatively, an attempt to produce more complex language not reflected in our other ELS measures. The change in dysfluencies was relatively small, however, and is not likely to be problematic in treatment studies. In addition, the Pearson correlations and intraclass correlations between scores on the repeated ELS administrations were significant for all measures in both conversation and narration for the two older age groups, demonstrating excellent test-retest reliability. In the youngest age group, the syntax and lexical diversity measures showed strong test-retest reliability in both conversation and narration. For the remaining ELS measures, the test-retest relationship was significant in one, but not both, of the sampling contexts for the youngest group. So, even for the 6- to 11-year-olds, the ELS procedures yield a choice of outcome measures with strong reproducibility whether one chooses to collect samples through conversation or narration in a treatment study.

The findings for construct validity were more variable across age groups. The 12- to 17-year-old group, which was the largest in terms of number of participants, yielded strong evidence of convergent validity for the ELS measures of syntax, lexical diversity, and unintelligibility. For the oldest group, we also found evidence of convergent validity for the syntax and unintelligibility measures; however, there was no evidence of construct validity for lexical diversity. Interestingly, for the oldest group, lexical diversity correlated significantly with several other validation measures (e.g., CELF-4 FS subtest and the SB5 VWM subtest), as well as with other ELS measures (e.g., syntax). This pattern of correlations raises the possibility that the ELS lexical diversity measure may index aspects of language other than the lexicon for this age group. Alternatively, the validation measure we selected (i.e., CELF-4 EV subtest) might not have been an ideal choice and may suffer from its own limitations in terms of construct validity. Although we favor the latter interpretation because the format of the CELF-4 EV is quite different from real-word social-linguistic interactions, deciding between these alternatives will require additional research and the inclusion of a different validation measure, preferably one differing in task demands from the CELF-4 EV subtest.

In contrast to the older groups, the findings for the 6- to 11-year-old group provided little support for the construct validity of the ELS-derived measures. Of the 10 correlations testing convergent validity (i.e., five measures in conversation and five in narration), only two approached statistical significance (syntax and dysfluency) and, then, only in a single sampling context. Testing these relationships, however, was complicated by the relatively small sample size and reduced statistical power for the youngest group. In keeping with this interpretation, the correlations between the validation measures and unintelligibility and dysfluency in conversation and lexical diversity, syntax, and unintelligibility in narration were all in the range of what would be described as of medium strength [73]. The lack of convergent validity may also be the result of limitations in the validation measures selected, particularly the test-like format of the standardized measures, which may result in these measures indexing more general cognitive processes (e.g., attention) and experiences rather than language-specific constructs. It may also be, however, that the relative lack of scaffolding (i.e., prompting, feedback, and reinforcement) built into the ELS procedures to ensure standardization made the conversational and narrative tasks too difficult for some younger participants. In this case, more play-based versions of expressive language sampling or book viewing with more examiner prompting might yield better results in terms of construct validity and compliance. We are pursuing this possibility in an ongoing project. For now, the present results suggest that the use of the specific ELS procedures evaluated in this project should be extended cautiously to young school-age participants in treatment studies, perhaps by ensuring that participants are somewhat higher functioning in language or cognition relative to the minimal inclusion/exclusion criteria we followed in this project.

#### Participant IQ

In contrast to the analyses focused on CA as a moderator of the psychometric properties of the ELS measures, the analyses focused on IQ showed little influence of this variable, at least as operationalized in the present study. Minimal practice effects and strong test-retest reliability were found for both the lower and the higher IQ group. In terms of construct validity, the ELS syntax and unintelligibility measures received strong support from our data for both the lower and the higher IQ group. The data for lexical diversity, however, contrasted with the findings for the full sample of participants, with no significant convergent validity correlations in conversation or narration for either IQ group. Again, this may reflect limitations of either the ELS lexical diversity measure or the CELF-4 EV subtest. The convergent validity correlations for ELS lexical diversity were especially low in the lower IQ group and in narration. Until further research on lexical diversity is conducted, it might be prudent to avoid using the ELS lexical diversity measure for lower IQ participants, use it only for conversational samples, or include it as a component of a composite score that also includes syntax and/or unintelligibility. In general, however, participant IQ need not be a major consideration when deciding on ELS as an outcome measure.

#### Participant ASD severity

ASD severity, operationalized as an ADOS-2 overall calibrated severity score in either (1) the minimal to mild range or (2) the moderate to severe range, did not exert a strong influence on the psychometric findings. Practice effects emerged only for the lower severity group for dysfluencies in narration, with dysfluencies becoming proportionally more frequent. Again, the effect for dysfluencies might reflect increased frustration with the repeated task or an attempt to produce more complex language that we did not detect. In any event, the change in dysfluencies was small and unlikely to create major interpretive difficulties within a treatment study. Test-retest correlations and intraclass correlations were statistically significant for all ELS-derived measures in conversation and in narration for both ASD severity groups. Syntax, lexical diversity, and unintelligibility in conversation and narration were significantly correlated with their respective validation measures for the higher ASD severity group. The construct validity correlations were less consistent for the lower ASD severity group, with only the ELS syntax measure meeting FDR criteria in both sampling contexts for that group; however, this would appear to reflect the relatively small sample size of this group given the absolute magnitude of the correlations for lexical diversity and unintelligibility. Thus, our findings suggest that there is little reason to consider ASD severity when deciding whether to use ELS measures as clinical endpoints.

### Limitations

It is important to recognize that there are many other approaches to expressive language sampling, including play-based methods developed for very young or minimally verbal children (e.g., [82, 83]) and the use of structured probes, as in the ADOS-2, to elicit specific types of language [84]. The present results do not necessarily extend to those other ELS procedures, which will need to be evaluated in terms of their psychometric adequacy as well. In addition, the present findings cannot be generalized beyond the type of participants with FXS included in the present sample, as all participants were capable of at least some multiword utterances according to parental report, although all had some level intellectual disability. In fact, the ELS procedures described here are unlikely to work well for more minimally verbal children, and thus, alternative methods for that important segment of the FXS population are needed [30]. Finally, it is important to note that we did not address the sensitivity to change of the ELS measures. The most direct way of addressing sensitivity would be to show that a treatment that is known to be efficacious in FXS leads to change in the ELS measures. As discussed at the outset, however, there are virtually no such treatments, although efficacious behavioral approaches are beginning to appear [85]. A less direct alternative, which we are currently pursuing, is to compare the magnitude of change in ELS-derived measures to that in standardized tests of the same constructs within the context of widely spaced longitudinal assessments during which change is highly likely. Combining this latter approach with the results of ELS measures used in treatment studies will be useful in making conclusions about sensitivity to change.

## Conclusions

The failure of more than two dozen clinical trials of medications for treating individuals with FXS has been attributed in part to the lack of psychometrically adequate outcome measures for this population. The present study was designed to evaluate the psychometric adequacy as outcome measures of five variables derived from a particular instantiation of ELS that includes standardization designed to increase consistency across administrations and minimize examiner influence on participant output. The findings suggest that the ELS procedures are feasible for a majority of 6 to 23 years with FXS who have intellectual disabilities. The measures derived in general are subject to minimal practice effects and display strong test-retest reliability. Three of the measures, reflecting vocabulary, syntax, and speech intelligibility, also have strong convergent validity, although their discriminant validity is limited. The ELS procedures and measures also work equally well for individuals regardless of their level of intellectual ability or degree of comorbid ASD severity. The procedures, however, are more challenging and have less adequate psychometric properties for those individuals with FXS under age 12, although the measures may still be useful for more linguistically and cognitive able children in this age range. Thus, the ELS measures studied in this paper promise to address an urgent need in the field of FXS.

## Supplementary information

**Additional file 1: Table S1.** Practice Effects Over a 4-Week Interval by Age Group. **Table S2.** Practice Effects Over a 4-Week Interval by IQ Group. **Table S3.** Practice Effects Over a 4-Week Interval by ASD Severity Group. **Table S4.** Test-Retest Reliability over a 4-Week Interval: Bivariate Correlations and Intraclass Correlations by Age Group. **Table S5.** Test-Retest Reliability over a 4-Week Interval: Bivariate Correlations and Intraclass Correlations by IQ Group. **Table S6.** Test-Retest Reliability over a 4-Week Interval: Bivariate Correlations and Intraclass Correlations by ASD Severity Group. **Table S7.** Construct Validity for Conversation by Age Group. **Table S8.** Construct Validity for Narration by Age Group. **Table S9.** Construct Validity for Conversation by IQ Group. **Table S10.** Construct Validity for Narration by IQ Group. **Table S11.** Construct Validity for Conversation by ASD Severity Group. **Table S12.** Construct Validity for Narration by ASD Severity Group.

## Data Availability

The datasets used and/or analyzed for the present paper can be made available upon a reasonable request to the corresponding author.

## Abbreviations

ID: Intellectual disability
FXS: Fragile X syndrome
ELS: Expressive language sampling
CA: Chronological age
SB5: Stanford-Binet Intelligence Scales, 5th edition
ADOS-2: Autism Diagnostic Observation Schedule, 2nd edition
SALT: Systematic Analysis of Language Transcripts
CELF-4: Clinical Evaluation of Language Fundamentals-4th edition
FS: Formulated Sentences subtest
EV: Expressive Vocabulary subtest
VWM: Verbal Working Memory subtest
GFTA: Goldman-Fristoe Test of Articulation
SiW: Single Words score
