# Correction to: Expressive language sampling as a source of outcome measures for treatment studies in fragile X syndrome: feasibility, practice effects, test-retest reliability, and construct validity

**DOI:** 10.1186/s11689-020-09314-5

**Published:** 2020-04-02

**Authors:** Leonard Abbeduto, Elizabeth Berry-Kravis, Audra Sterling, Stephanie Sherman, Jamie O. Edgin, Andrea McDuffie, Anne Hoffmann, Debra Hamilton, Michael Nelson, Jeannie Aschkenasy, Angela John Thurman

**Affiliations:** 1 UC Davis MIND Institute and Department of Psychiatry and Behavioral Sciences, University of California, 2825 50th St. Davis, Sacramento, CA 95817 USA; 2 Departments of Pediatrics, Neurological Sciences and Biochemistry, Rush University Medical Center, Chicago, USA; 3 Waisman Center and Department of Communication Sciences and Disorders, University of Wisconsin-Madison, Madison, USA; 4 Department of Human Genetics, Emory University, Atlanta, USA; 5 Department of Psychology, University of Arizona, Tucson, USA

**Correction to: J Neurodevelop Disord (2020) 12:10**

**https://doi.org/10.1186/s11689-020-09313-6**

In the original publication of this article [1], the author name Leonard Abbeduto was misspelled as Leonardkk Abbeduto. The original article has been corrected.

The publisher apologizes for the inconvenience this has caused.
