# Supplementary material for: Expressive language sampling as a source of outcome measures for treatment studies in fragile X syndrome: feasibility, practice effects, test-retest reliability, and construct validity
Source: J Neurodev Disord. 2020 Mar 24;12:10. doi: 10.1186/s11689-020-09313-6 (PMC7092603; doi:10.1186/s11689-020-09313-6)
Supplement: Supplementary file 1 — Additional file 1: Table S1. Practice Effects Over a 4-Week Interval by Age Group. Table S2. Practice Effects Over a 4-Week Interval by IQ Group. Table S3. Practice Effects Over a 4-Week Interval by ASD Severity Group. Table S4. Test-Retest Reliability over a 4-Week Interval: Bivariate Correlations and Intraclass Correlations by Age Group. Table S5. Test-Retest Reliability over a 4-Week Interval: Bivariate Correlations and Intraclass Correlations by IQ Group. Table S6. Test-Retest Reliability over a 4-Week Interval: Bivariate Correlations and Intraclass Correlations by ASD Severity Group. Table S7. Construct Validity for Conversation by Age Group. Table S8. Construct Validity for Narration by Age Group. Table S9. Construct Validity for Conversation by IQ Group. Table S10. Construct Validity for Narration by IQ Group. Table S11. Construct Validity for Conversation by ASD Severity Group. Table S12. Construct Validity for Narration by ASD Severity Group. [file 11689_2020_9313_MOESM1_ESM.docx]

**Supplementary Tables**

Supplementary Table 1. Practice Effects Over a 4-Week Interval by Age Group

| **Measure** | **Conversation** | | | | **Narration** | | | |
| --- | --- | --- | --- | --- | --- | --- | --- | --- |
|  | **Visit 1** | | **Retest** | | **Visit 1** | | **Retest** | |
|  | ***M*** | ***SD*** | ***M*** | ***SD*** | ***M*** | ***SD*** | ***M*** | ***SD*** |
| **6- to 11-year-olds (n = 17-18)** | | | | | | | | |
| **Lexical Diversity** | 67.17 | 19.03 | 69.11 | 25.02 | 58.41 | 24.57 | 58.76 | 21.02 |
| **Syntax** | 3.09 | 0.92 | 3.23 | 0.95 | 3.77 | 1.15 | 3.53 | 0.95 |
| **Talkativeness** | 14.47 | 4.24 | 16.14 | 5.74 | 11.54 | 4.39 | 13.58 | 5.34 |
| **Unintelligibility** | 0.21 | 0.10 | 0.20 | 0.11 | 0.26 | 0.17 | 0.26 | 0.20 |
| **Dysfluency** | 0.19 | 0.11 | 0.16 | 0.09 | 0.17 | 0.13 | 0.17 | 0.08 |
| **12- to 17-year-olds (n = 47-49)** | | | | | | | | |
| **Lexical Diversity** | 81.96 | 36.37 | 87.83 | 36.85 | 71.51 | 32.83 | 73.90 | 32.24 |
| **Syntax** | 3.93 | 1.55 | 4.11 | 1.56 | 5.00 | 2.07 | 5.31 | 2.22 |
| **Talkativeness** | 14.20 | 6.22 | 13.96 | 4.89 | 12.55 | 5.77 | 11.96 | 4.84 |
| **Unintelligibility** | 0.12 | 0.11 | 0.13 | 0.13 | 0.12 | 0.17 | 0.14 | 0.16 |
| **Dysfluency** | 0.22 | 0.15 | 0.23 | 0.14 | 0.17*^a^ | 0.12 | 0.20*^a^ | 0.15 |
| **18- to 23-year-olds (n = 22)** | | | | | | | | |
| **Lexical Diversity** | 78.37 | 28.46 | 81.82 | 32.22 | 65.55 | 33.52 | 68.23 | 37.19 |
| **Syntax** | 3.48 | 1.22 | 3.64 | 1.44 | 5.07 | 2.68 | 5.23 | 2.68 |
| **Talkativeness** | 15.40 | 4.37 | 15.64 | 4.20 | 12.51 | 5.88 | 11.47 | 5.57 |
| **Unintelligibility** | 0.16 | 0.18 | 0.18 | 0.21 | 0.15 | 0.17 | 0.14 | 0.13 |
| **Dysfluency** | 0.19 | 0.11 | 0.19 | 0.12 | 0.16*^a^ | 0.13 | 0.21*^a^ | 0.15 |

Uncorrected *p* values for individual tests comparing means for test and retest administrations are marked with asterisks as follows: *** signifies *p* < .0005. ** signifies *p* < .005. ** signifies *p* < .050. No comparison was significant at *p* < .050 after correcting for multiple tests through the FDR procedure. ^a^ *p* > .050 in Wilcoxon Signed Ranks (nonparametric) analysis.  ^b^ *p* < .050 in Spearman (nonparametric) analysis.

Supplementary Table 2. Practice Effects Over a 4-Week Interval by IQ Group

| **Measure** | **Conversation** | | | | **Narration** | | | |
| --- | --- | --- | --- | --- | --- | --- | --- | --- |
|  | **Visit 1** | | **Retest** | | **Visit 1** | | **Retest** | |
|  | ***M*** | ***SD*** | ***M*** | ***SD*** | ***M*** | ***SD*** | ***M*** | ***SD*** |
| **Lower IQ (n = 40)** | | | | | | | | |
| **Lexical Diversity** | 62.35 | 26.32 | 67.48 | 28.07 | 48.05 | 20.29 | 49.15 | 19.40 |
| **Syntax** | 2.92 | 0.97 | 3.04 | 0.97 | 3.62 | 1.30 | 3.81 | 1.36 |
| **Talkativeness** | 14.09 | 5.62 | 14.97 | 5.40 | 11.46 | 5.17 | 11.52 | 5.05 |
| **Unintelligibility** | 0.19 | 0.14 | 0.21 | 0.17 | 0.21 | 0.17 | 0.21 | 0.18 |
| **Dysfluency** | 0.15 | 0.09 | 0.15 | 0.08 | 0.13 | 0.11 | 0.14 | 0.10 |
| **Higher IQ (n = 39-41)** | | | | | | | | |
| **Lexical Diversity** | 93.63 | 30.38 | 97.32 | 33.61 | 89.72 | 25.64 | 90.95 | 28.84 |
| **Syntax** | 4.33 | 1.41 | 4.56 | 1.42 | 6.23 | 2.10 | 6.34 | 2.28 |
| **Talkativeness** | 14.43 | 5.02 | 14.52 | 4.74 | 12.67 | 5.25 | 12.25 | 4.86 |
| **Unintelligibility** | 0.09 | 0.09 | 0.08 | 0.08 | 0.08 | 0.11 | 0.09 | 0.11 |
| **Dysfluency** | 0.25 | 0.14 | 0.26 | 0.13 | 0.23 | 0.12 | 0.25 | 0.16 |

No comparison of means for test and retest administrations was significant at *p* < .050 (even when uncorrected for multiple tests).

Supplementary Table 3. Practice Effects Over a 4-Week Interval by ASD Severity Group

| **Measure** | **Conversation** | | | | **Narration** | | | |
| --- | --- | --- | --- | --- | --- | --- | --- | --- |
|  | **Visit 1** | | **Retest** | | **Visit 1** | | **Retest** | |
|  | ***M*** | ***SD*** | ***M*** | ***SD*** | ***M*** | ***SD*** | ***M*** | ***SD*** |
| **Less Severe ASD Symptoms (*n* = 26)** | | | | | | | | |
| **Lexical Diversity** | 98.23 | 27.73 | 101.456 | 32.80 | 84.04 | 33.33 | 89.31 | 34.46 |
| **Syntax** | 4.67 | 1.38 | 4.76 | 1.48 | 5.93 | 2.26 | 6.27 | 2.38 |
| **Talkativeness** | 15.47 | 5.22 | 15.93 | 5.53 | 11.71 | 4.17 | 11.05 | 3.87 |
| **Unintelligibility** | .08 | .08 | .09 | .08 | .08 | .10 | .08 | .09 |
| **Dysfluency** | .29 | .16 | .29 | .13 | .23** | .14 | .30** | .17 |
| **More Severe ASD Symptoms (*n* = 55 - 56)** | | | | | | | | |
| **Lexical Diversity** | 68.85 | 29.50 | 75.04.55 | 32.55 | 59.68 | 28.87 | 61.11 | 25.18 |
| **Syntax** | 3.21 | 1.19 | 3.41 | 1.31 | 4.19 | 1.96 | 4.33 | 1.93 |
| **Talkativeness** | 14.55 | 5.23 | 14.81 | 4.40 | 12.93 | 6.12 | 12.68 | 5.47 |
| **Unintelligibility** | .18 | .14 | .19 | .17 | .19 | .19 | .20 | .18 |
| **Dysfluency** | .17 | .10 | .17 | .11 | .14 | .10 | .15 | .10 |

Uncorrected *p* values for individual tests comparing means for test and retest administrations are marked with asterisks as follows: *** signifies *p* < .0005. ** signifies *p* < .005. ** signifies *p* < .050. Shaded cells contain values that were significant at *p* < .050 after correcting for multiple tests through the FDR procedure.

Supplementary Table 4. Test-Retest Reliability over a 4-Week Interval: Bivariate Correlations and Intraclass Correlations by Age Group

| **Measure** | **Conversation** | | **Narration** | |
| --- | --- | --- | --- | --- |
|  | ***r*** | ***icc*** | ***r*** | ***icc*** |
| **6- to 11-year-olds (n = 17-18)** | | | | |
| **Lexical Diversity** | .53*^a^ | .69* | .71** | .83** |
| **Syntax** | .56* | .72** | .72** | .82** |
| **Talkativeness** | .52*^a^ | .65* | .35 | .49 |
| **Unintelligibility** | .22 | .38 | .75** | .86*** |
| **Dysfluency** | .46^b^ | .62* | .50* | .60* |
| **12- to 17-year-olds (n = 47-49)** | | | | |
| **Lexical Diversity** | .72*** | .83*** | .90*** | .95*** |
| **Syntax** | .86*** | .92*** | .86*** | .92*** |
| **Talkativeness** | .80*** | .88*** | .80*** | .88*** |
| **Unintelligibility** | .78*** | .86*** | .72*** | .84*** |
| **Dysfluency** | .76*** | .86*** | .76*** | .84*** |
| **18- to 23-year-olds (n = 22)** | | | | |
| **Lexical Diversity** | .91*** | .95*** | .88*** | .94*** |
| **Syntax** | .92*** | .95*** | .93*** | .96*** |
| **Talkativeness** | .81*** | .90*** | .76*** | .86*** |
| **Unintelligibility** | .97*** | .98*** | .81*** | .88*** |
| **Dysfluency** | .87*** | .93*** | .83*** | .88*** |

Note that uncorrected *p* values for individual tests are marked with asterisks as follows: *** signifies *p* < .0005. ** signifies *p* < .005. ** signifies *p* < .050. Shaded cells contain values that were significant at p < .050 after correcting for multiple tests through the FDR procedure. ^a^ *p* > .050 in Spearman (nonparametric) analysis.  ^b^ *p* < .050 in Spearman (nonparametric) analysis.Supplementary Table 5. Test-Retest Reliability over a 4-Week Interval: Bivariate Correlations and Intraclass Correlations by IQ Group

| **Measure** | **Conversation** | | **Narration** | |
| --- | --- | --- | --- | --- |
|  | ***r*** | ***icc*** | ***r*** | ***icc*** |
| **Lower IQ (n = 40)** | | | | |
| **Lexical Diversity** | .63*** | .77*** | .73*** | .84*** |
| **Syntax** | .71*** | .83*** | .82*** | .90*** |
| **Talkativeness** | .71*** | .82*** | .60*** | .76*** |
| **Unintelligibility** | .77*** | .86*** | .78*** | .88*** |
| **Dysfluency** | .46** | .63** | .44**  88 | .62** |
| **Higher IQ (n = 39-41)** | | | | |
| **Lexical Diversity** | .71*** | .83*** | .80*** | .89*** |
| **Syntax** | .83*** | .90*** | .82*** | .90*** |
| **Talkativeness** | .79*** | .88*** | .76*** | .86*** |
| **Unintelligibility** | .79*** | .88*** | .66*** | .80*** |
| **Dysfluency** | .76*** | .86*** | .82*** | .88*** |

Note that uncorrected *p* values for individual tests are marked with asterisks as follows: *** signifies *p* < .0005. ** signifies *p* < .005. ** signifies *p* < .050. Shaded cells contain values that were significant at *p* < .050 after correcting for multiple tests through the FDR procedure.

Supplementary Table 6. Test-Retest Reliability over a 4-Week Interval: Bivariate Correlations and Intraclass Correlations by ASD Severity Group

|  | **Conversation** | | **Narration** | |
| --- | --- | --- | --- | --- |
| **Measure** | ***r*** | ***icc*** | ***r*** | ***icc*** |
| **Lower ASD Severity (*n* = 26)** | | | | |
| **Lexical Diversity** | .70*** | .82*** | .83*** | .91*** |
| **Syntax** | .83*** | .91*** | .86*** | .92*** |
| **Talkativeness** | .80*** | .89*** | .62** | .77*** |
| **Unintelligibility** | .76*** | .87*** | .61** | .76*** |
| **Dysfluency** | .65*** | .79*** | .78*** | .84*** |
| **Higher ASD Severity (*n* = 55 - 56)** | | | | |
| **Lexical Diversity** | .71*** | .82*** | .88*** | .94*** |
| **Syntax** | .80*** | .88*** | .86*** | .92*** |
| **Talkativeness** | .63*** | .77*** | .70*** | .83*** |
| **Unintelligibility** | .80*** | .88*** | .74*** | .85*** |
| **Dysfluency** | .72*** | .83*** | .57*** | .73*** |

Note that uncorrected *p* values for individual tests are marked with asterisks as follows: *** signifies *p* < .0005. ** signifies *p* < .005. ** signifies *p* < .050. Shaded cells contain values that were significant at *p* < .050 after correcting for multiple tests through the FDR procedure.

Supplementary Table 7. Construct Validity for Conversation by Age Group

| **Measure** | **CELF**  **EV** | **CELF**  **FS** | **Vineland**  **EC** | **GFTA**  **SiW** | **SB5**  **VWM** |
| --- | --- | --- | --- | --- | --- |
| **6- to 11-year-olds (**n **= 15-18)** | | | | | |
| **Lexical**  **Diversity** | **-.21^b^** | .10 | .16 | .19 | .11 |
| **Syntax** | -.13 | **.12** | .24 | .47*^a^ | .18 |
| **Talkativeness** | -.11 | .09 | **-.03** | .10 | .06 |
| **Unintelligibility** | -.02 | -.10 | -.14 | **-.31** | .06 |
| **Dysfluency** | -.11 | -.07 | -.20 | .21 | **-.48** |
| **12- to 17-year-olds (*n* = 43-47)** | | | | | |
| **Lexical**  **Diversity** | **.51***** | .63*** | .29 | .45** | .47** |
| **Syntax** | .50*** | **.70***** | .34* | .48** | .51*** |
| **Talkativeness** | -.09 | -.06 | **.02** | -.08 | -.18 |
| **Unintelligibility** | .38* | .48** | -.36* | **-.65***** | -.49** |
| **Dysfluency** | .42** | .57*** | .36* | .31* | **.07** |
| **18- to 23-year-olds (*n* = 19-22)** | | | | | |
| **Lexical**  **Diversity** | **.37** | .68*** | .43 | .52* | .58** |
| **Syntax** | .47* | **.69***** | .41 | .61** | .67** |
| **Talkativeness** | .13 | .21 | **.17** | -.06 | .22 |
| **Unintelligibility** | .01 | -.54* | -.23 | **-.70***** | -.25 |
| **Dysfluency** | .50* | .72*** | .57* | .52* | **.02** |

Note that all values are bivariate zero-order correlations except for that between dysfluency and the SB5 VWM score, which is a partial correlation controlling for syntax (MLU). Uncorrected *p* values for individual tests are marked with asterisks as follows: *** signifies *p* < .0005. ** signifies *p* < .005. ** signifies *p* < .050. Shaded cells contain values that were significant at p < .050 after correcting for multiple tests through the FDR procedure. The boldfaced values represent convergent validity relationships; all other values represent discriminant validity relationships. ^a^ *p* > .050 in Spearman (nonparametric) analysis.  ^b^ *p* < .050 in Spearman (nonparametric) analysis.

Supplementary Table 8. Construct Validity for Narration by Age Group

| **Measure** | **CELF**  **EV** | **CELF**  **FS** | **Vineland**  **EC** | **GFTA**  **SiW** | **SB5**  **VWM** |
| --- | --- | --- | --- | --- | --- |
| **6- to 11-year-olds (*n* = 14-17)** | | | | | |
| **Lexical**  **Diversity** | .**31** | .27 | .21 | .57* | .44 |
| **Syntax** | .40 | **.45** | .18 | .66** | .66** |
| **Talkativeness** | -.16 | .09 | -**.24** | -.19 | .00 |
| **Unintelligibility** | -.06 | -.08 | -.47 | **-.37^b^** | -.31 |
| **Dysfluency** | .05 | .16 | -.18 | .34 | **-.16** |
| **12- to 17-year-olds (*n* = 46-50)** | | | | | |
| **Lexical**  **Diversity** | **.50***** | .50*** | .37* | .50*** | .56*** |
| **Syntax** | .56*** | **.76***** | .42** | .54*** | .75*** |
| **Talkativeness** | -.08 | -.19 | **-.03** | -.05 | -.23 |
| **Unintelligibility** | -.33* | -.44** | -.38* | **-.51***** | -.47** |
| **Dysfluency** | .41** | .54*** | .17 | .27^b^ | **-.13** |
| **18- to 23-year-olds (*n* = 19-22)** | | | | | |
| **Lexical**  **Diversity** | **.14** | -.67** | .52* | .45* | .73*** |
| **Syntax** | .40 | **.85***** | .53* | .57* | .80*** |
| **Talkativeness** | -.02 | -.05 | **.06** | -.28 | -.02 |
| **Unintelligibility** | .07 | -.49* | -.60* | **-.63**** | -.34 |
| **Dysfluency** | .05 | .54* | .68** | .36 | **.08** |

Note that all values are bivariate zero-order correlations except for that between dysfluency and the SB5 VWM score, which is a partial correlation controlling for syntax (MLU). Uncorrected *p* values for individual tests are marked with asterisks as follows: *** signifies *p* < .0005. ** signifies *p* < .005. ** signifies *p* < .050. Shaded cells contain values that were significant at p < .050 after correcting for multiple tests through the FDR procedure. The boldfaced values represent convergent validity relationships; all other values represent discriminant validity relationships. ^a^ *p* > .050 in Spearman (nonparametric) analysis.  ^b^ *p* < .050 in Spearman (nonparametric) analysis.

Supplementary Table 9. Construct Validity for Conversation by IQ Group

| **Measure** | **CELF**  **EV** | **CELF**  **FS** | **Vineland**  **EC** | **GFTA**  **SiW** | **SB5**  **VWM** |
| --- | --- | --- | --- | --- | --- |
| **Lower IQ (*n* = 37 - 40)** | | | | | |
| **Lexical**  **Diversity** | **.21** | .50** | .08 | .34*^a^ | .36* |
| **Syntax** | .29 | **.50**** | .14 | .46** | .51** |
| **Talkativeness** | -.11 | .09 | **.05** | -.05 | -.02 |
| **Unintelligibility** | -.05 | -.32*^a^ | -.09 | **-.42*** | .04 |
| **Dysfluency** | .20 | .23 | .27^b^ | .22 | **-.20** |
| **Higher IQ (*n* = 37- 41)** | | | | | |
| **Lexical**  **Diversity** | **.32*** | .49** | .26 | .28^b^ | .21 |
| **Syntax** | .33* | **.57***** | .33* | .40* | .28 |
| **Talkativeness** | -.18 | .00 | **-.13** | -.08 | -.30 |
| **Unintelligibility** | -.09^b^ | -.48** | -.59*** | **-.68***** | -.50** |
| **Dysfluency** | .30^b^ | .48* | .25 | .15 | **.04** |

Note that all values are bivariate zero-order correlations except for that between dysfluency and the SB5 VWM score, which is a partial correlation controlling for syntax (MLU). Uncorrected *p* values for individual tests are marked with asterisks as follows: *** signifies *p* < .0005. ** signifies *p* < .005. ** signifies *p* < .050. Shaded cells contain values that were significant at p < .050 after correcting for multiple tests through the FDR procedure. The boldfaced values represent convergent validity relationships; all other values represent discriminant validity relationships. ^a^ *p* > .050 in Spearman (nonparametric) analysis.  ^b^ *p* < .050 in Spearman (nonparametric) analysis.

Supplementary Table 10. Construct Validity for Narration by IQ Group

| **Measure** | **CELF**  **EV** | **CELF**  **FS** | **Vineland**  **EC** | **GFTA**  **SiW** | **SB5**  **VWM** |
| --- | --- | --- | --- | --- | --- |
| **Lower IQ** **(*n* = 37 - 40)** | | | | | |
| **Lexical**  **Diversity** | **-.03** | .21 | .14 | .29 | .33* |
| **Syntax** | .33*^a^ | **.53***** | .24 | .53*** | .58*** |
| **Talkativeness** | -.19 | -.09 | **-.04** | -.13 | -.07 |
| **Unintelligibility** | -.14 | -.26 | -.26^b^ | **-.34*** | -.12 |
| **Dysfluency** | -.09 | .04 | .28 | .08 | **-.08** |
| **Higher IQ (*n* = 37 - 40)** | | | | | |
| **Lexical**  **Diversity** | **.19** | .37* | .13 | .35* | .30 |
| **Syntax** | .37* | **.74***** | .24 | .35* | .66*** |
| **Talkativeness** | -.02 | -.34* | **-.10** | -.19 | -.49** |
| **Unintelligibility** | -.04 | -.36* | -.61*** | **-.53**^a^** | -.46** |
| **Dysfluency** | .28^b^ | .45** | .02 | .18 | **-.09** |

Note that all values are bivariate zero-order correlations except for that between dysfluency and the SB5 VWM score, which is a partial correlation controlling for syntax (MLU). Uncorrected *p* values for individual tests are marked with asterisks as follows: *** signifies *p* < .0005. ** signifies *p* < .005. ** signifies *p* < .050. Shaded cells contain values that were significant at p < .050 after correcting for multiple tests through the FDR procedure. The boldfaced values represent convergent validity relationships; all other values represent discriminant validity relationships. ^a^ *p* > .050 in Spearman (nonparametric) analysis.  ^b^ *p* < .050 in Spearman (nonparametric) analysis.

Supplementary Table 11. Construct Validity for Conversation by ASD Severity Group

| **Measure** | **CELF**  **EV** | **CELF**  **FS** | **Vineland**  **EC** | **GFTA**  **SiW** | **SB5**  **VWM** |
| --- | --- | --- | --- | --- | --- |
| **Lower ASD Symptom Severity (*n* = 23 - 27)** | | | | | |
| **Lexical Diversity**  **Diversity** | **.24** | .45* | .40* | .24^b^ | .35 |
| **Syntax** | .33 | **.55**** | .21 | .28 | .39* |
| **Talkativeness** | -.41*^a^ | -.25 | **-.18^b^** | -.31 | -.21 |
| **Unintelligibility** | -.42*^a^ | -.41*^a^ | -.32^b^ | **-.70***^a^** | -.46* |
| **Dysfluency** | .25 | .45* | .22 | .18 | **.01** |
| **Higher ASD Symptom Severity (*n* = 51-55)** | | | | | |
| **Lexical Diversity**  **Diversity** | .**44**** | .62*** | .19 | .42** | .39** |
| **Syntax** | .45** | **.59***** | .38** | .51*** | .47*** |
| **Talkativeness** | .03 | .10 | **.25** | .00 | -.03 |
| **Unintelligibility** | -.06 | -.43** | .25^b^ | **-.50***** | -.26 |
| **Dysfluency** | .34* | .40** | 35* | .30* | **-.19** |

Note that all values are bivariate zero-order correlations except for that between dysfluency and the SB5 VWM score, which is a partial correlation controlling for syntax (MLU). Uncorrected *p* values for individual tests are marked with asterisks as follows: *** signifies *p* < .0005. ** signifies *p* < .005. ** signifies *p* < .050. Shaded cells contain values that were significant at p < .050 after correcting for multiple tests through the FDR procedure. The boldfaced values represent convergent validity relationships; all other values represent discriminant validity relationships. ^a^ *p* > .050 in Spearman (nonparametric) analysis.  ^b^ *p* < .050 in Spearman (nonparametric) analysis.

Supplementary Table 12. Construct Validity for Narration by ASD Severity Group

| **Measure** | **CELF**  **EV** | **CELF**  **FS** | **Vineland**  **EC** | **GFTA**  **SiW** | **SB5**  **VWM** |
| --- | --- | --- | --- | --- | --- |
| **Lower ASD Symptom Severity (*n* = 24 - 27)** | | | | | |
| **Lexical Diversity**  **Diversity** | .42* | .52* | .35 | .54** | .60** |
| **Syntax** | .70*** | **.79***** | .40* | .51* | .68*** |
| **Talkativeness** | -.16 | -.14 | **.08** | .11 | .04 |
| **Unintelligibility** | -.40*^a^ | -.51* | -.65*** | **-.33** | -.46* |
| **Dysfluency** | .31 | .54** | .27 | .29 | **-.06** |
| **Higher ASD Symptom Severity** **(*n* = 52 – 56)** | | | | | |
| **Lexical Diversity**  **Diversity** | .37** | .59*** | .35* | .50*** | .54*** |
| **Syntax** | .43** | **.75***** | .34* | .50*** | .54*** |
| **Talkativeness** | .02 | -.01 | **.06** | -.05 | -.15 |
| **Unintelligibility** | -.22^b^ | -.40** | -.29* | **-.48***** | -.36* |
| **Dysfluency** | .13 | .25^b^ | .28* | .10^b^ | **-.21** |

Note that all values are bivariate zero-order correlations except for that between dysfluency and the SB5 VWM score, which is a partial correlation controlling for syntax (MLU-C). *** signifies *p* < .0005. ** signifies *p* < .005. ** signifies *p* < .050. Shaded cells contain values that were significant at *p* < .050 after correcting for multiple tests through the FDR procedure. The boldfaced values represent convergent validity relationships; all other values represent discriminant validity relationships. ^a^ *p* > .050 in Spearman (nonparametric) analysis.  ^b^ *p* < .050 in Spearman (nonparametric) analysis.
